# Functional screening reveals *Toxoplasma* prenylated proteins required for endocytic trafficking and rhoptry protein sorting

**DOI:** 10.1128/mbio.01309-23

**Published:** 2023-08-07

**Authors:** Qiang-Qiang Wang, Ming Sun, Tao Tang, De-Hua Lai, Jing Liu, Sanjay Maity, Kai He, Xi-Ting Wu, Jiong Yang, Yue-Bao Li, Xiao-Yan Tang, Hui-Yong Ding, Geoff Hide, Mark Distefano, Zhao-Rong Lun, Xing-Quan Zhu, Shaojun Long

**Affiliations:** 1 National Key Laboratory of Veterinary Public Health Security and College of Veterinary Medicine, China Agricultural University, Beijing, China; 2 National Animal Protozoa Laboratory and School of Veterinary Medicine, China Agricultural University, Beijing, China; 3 MOE Key Laboratory of Gene Function and Regulation, State Key Laboratory of Biocontrol, School of Life Sciences, Sun Yat-sen University, Guangzhou, China; 4 Department of Chemistry, University of Minnesota, Minneapolis, Minnesota, USA; 5 Department of Medicinal Chemistry, University of Minnesota, Minneapolis, Minnesota, USA; 6 Biomedical Research and Innovation Centre and Environmental Research and Innovation Centre, School of Science, Engineering and Environment, University of Salford, Salford, United Kingdom; 7 College of Veterinary Medicine, Shanxi Agricultural University, Jinzhong, Shanxi Province, China; Albert Einstein College of Medicine, Bronx, New York, USA

**Keywords:** apicomplexans, *Toxoplasma gondii*, malaria parasites, protein prenylation, prenylome, endocytic trafficking, digestive vacuole, secretory trafficking, rhoptry biogenesis

## Abstract

In the apicomplexans, endocytosed cargos (e.g., hemoglobin) are trafficked to a specialized organelle for digestion. This follows a unique endocytotic process at the micropore/cytostome in these parasites. However, the mechanism underlying endocytic trafficking remains elusive, due to the repurposing of classical endocytic proteins for the biogenesis of apical organelles. To resolve this issue, we have exploited the genetic tractability of the model apicomplexan *Toxoplasma gondii*, which ingests host cytosolic materials (e.g., green fluorescent protein[GFP]). We determined an association between protein prenylation and endocytic trafficking, and using an alkyne-labeled click chemistry approach, the prenylated proteome was characterized. Genome editing, using clustered regularly interspaced short palindromic repaet/CRISPR-associated nuclease 9 (CRISPR/Cas9), was efficiently utilized to generate genetically modified lines for the functional screening of 23 prenylated candidates. This identified four of these proteins that regulate the trafficking of endocytosed GFP vesicles. Among these proteins, Rab1B and YKT6.1 are highly conserved but are non-classical endocytic proteins in eukaryotes. Confocal imaging analysis showed that Rab1B and Ras are substantially localized to both the trans-Golgi network and the endosome-like compartments in the parasite. Conditional knockdown of Rab1B caused a rapid defect in secretory trafficking to the rhoptry bulb, suggesting a trafficking intersection role for the key regulator Rab1B. Further experiments confirmed a critical role for protein prenylation in regulating the stability/activity of these proteins (i.e., Rab1B and YKT6.1) in the parasite. Our findings define the molecular basis of endocytic trafficking and reveal a potential intersection function of Rab1B on membrane trafficking in *T. gondii*. This might extend to other related protists, including the malarial parasites.

**IMPORTANCE:**

The protozoan *Toxoplasma gondii* establishes a permissive niche, in host cells, that allows parasites to acquire large molecules such as proteins. Numerous studies have demonstrated that the parasite repurposes the classical endocytic components for secretory sorting to the apical organelles, leaving the question of endocytic transport to the lysosome-like compartment unclear. Recent studies indicated that endocytic trafficking is likely to associate with protein prenylation in malarial parasites. This information promoted us to examine this association in the model apicomplexan *T. gondii* and to identify the key components of the prenylated proteome that are involved. By exploiting the genetic tractability of *T. gondii* and a host GFP acquisition assay, we reveal four non-classical endocytic proteins that regulate the transport of endocytosed cargos (e.g., GFP) in *T. gondii*. Thus, we extend the principle that protein prenylation regulates endocytic trafficking and elucidate the process of non-classical endocytosis in *T. gondii* and potentially in other related protists.

## INTRODUCTION

*Toxoplasma gondii* is an obligate intracellular parasite that resides within a limiting membrane called the parasitophorous vacuole membrane (PVM). *T. gondii* belongs to the subphylum Apicomplexa, which also includes other notorious pathogens such as *Plasmodium*, the causative agents of human malaria. Membrane trafficking functions in both the biogenesis and digestion processes in *T. gondii* (1). These processes involve familiar structures found widely in eukaryotes but also specialized organelles that include the secretory organelles, the micropore, and lysosome-like organelles. Endocytosis in this parasite operates by an analogous pathway that has been recently discovered at the micropore in *T. gondii* (2, 3). This process is able to generate membrane homeostasis (2) and to acquire host cytosolic materials for nutrient acquisition (3). These cargos are internalized by invagination of the parasite plasma membrane and trafficked to the vacuolar compartment (VAC)/plant-like vacuole compartment for digestion (4 - 7).

The ability to deliver host cytosol-derived material to the VAC for digestion contributes to the acute stage infection in *T. gondii* (3, 4). However, it remains unclear how the endocytosed cargos are transported to the digestive vacuole. Recent studies have discovered that the only classical protein, involved in the process in *Plasmodium falciparum* and *T. gondii*, is VPS45, a Sec/Munc18 protein (8, 9). Inactivation of this protein caused diffusion of vesicles containing hemoglobin in *P. falciparum* and retention of GFP vesicles in the endosomes in *T. gondii*. This vesicle-dependent process appears to be akin to the endocytic trafficking mechanism in the model organisms of mammals and yeast cells. It is therefore conceivable that this endocytic trafficking process, in these early divergent eukaryotes, could have involved endosome formation, endosome specificity, and membrane fusion, similar to that seen in the model organisms (10, 11).

In *T. gondii*, all other studied protein homologs, that traditionally participate in the endolysosomal pathway, are involved in secretory trafficking for protein sorting and biogenesis of specialized secretory organelles (i.e., micronemes, rhoptries, and dense granules) (12 - 14). These proteins include DrpB (15), sortilin (16), Rab5A, Rab5C (17), Rab7 (18), retromer proteins (19), class C core vacuole/endosome tethering, and homotypic fusion and vacuole protein sorting (18, 20, 21). The secretory organelles, in the phylum Apicomplexa, are crucial for cell invasion and intracellular survival of these parasites and are, therefore, under significant evolutionary selection pressure to function effectively (1). Repurposing of components of the classical endocytic complexes would therefore be vital for *de novo* biogenesis of the secretory organelles used for intracellular parasitism and pathogenesis. With the exception of those components used in the repurposing model, some apicomplexans appear to have lost other recognizable endocytic trafficking complexes, including some or all components of the endosomal sorting complexes required for transport 0/I/II and Golgi-localized, gamma adaptin ear-containing, adenosine diphosphate-ribosylation factor (ARF)-binding proteins (14, 22, 23). These observations potentially suggest the absence of a functional endocytic system or one which is highly divergent. However, endocytic structures, such as the cytostome/micropore (2, 3, 24), lysosome-like vacuoles (25, 26), and endosomal-like compartments (27) exist and are active in the parasites, as discussed above. Therefore, it leaves the enigmatic question of which proteins govern the steps in endocytic transport for the digestion of endocytosed cargos in the digestive vacuole. Research in this direction would further our understanding of the endocytic and secretory trafficking systems that ensure successful parasite nutrient acquisition and organelle biogenesis during infections.

In *P. falciparum,* drug inhibition of apicoplast isoprenoid biosynthesis caused defects in hemoglobin transport and the digestive vacuole (28, 29), providing valuable clues as to the nature of endocytic trafficking regulation. Protein prenylation occurs by the covalent addition of two types of isoprenoids, farnesyl pyrophosphate (FPP) (C15) or geranylgeranyl pyrophosphate (GGPP) (C20), to amino acid sequences involving cysteine residues, such as -CaaX, -CC, or -CxC, at or near the carboxyl terminus (30). This modification has been recognized as a key physiological process for facilitating membrane-associated protein trafficking. However, the regulatory mechanisms, involving isoprenoids, affecting hemoglobin transport in *P. falciparum* and endocytic transport in other related parasites have still remained elusive.

To resolve this issue, we have exploited the genetic tractability of the apicomplexan model organism *T. gondii*, which has a process enabling the uptake of host cytosolic material (e.g., GFP). We have determined an association between protein prenylation and the trafficking pathway using drug inhibition of isoprenoid biosynthesis and genetic studies on prenyltransferases. From the prenylated proteomics candidates, identified by an alkyne-labeled click chemistry strategy in this study, our functional screening successfully identified four non-classical endocytic regulators, among which Rab1B and YKT6.1 are highly conserved in the apicomplexans. Our detailed analyses revealed the molecular basis for endocytic trafficking and the regulatory function of isoprenoids in *T. gondii*. These studies, in the model organism *T. gondii*, provide a basis for extending this knowledge to other apicomplexan pathogens including the malarial parasites.

## RESULTS

### Prenylated proteins are likely to be involved in the trafficking of endocytosed cargos

**Fig 1:**
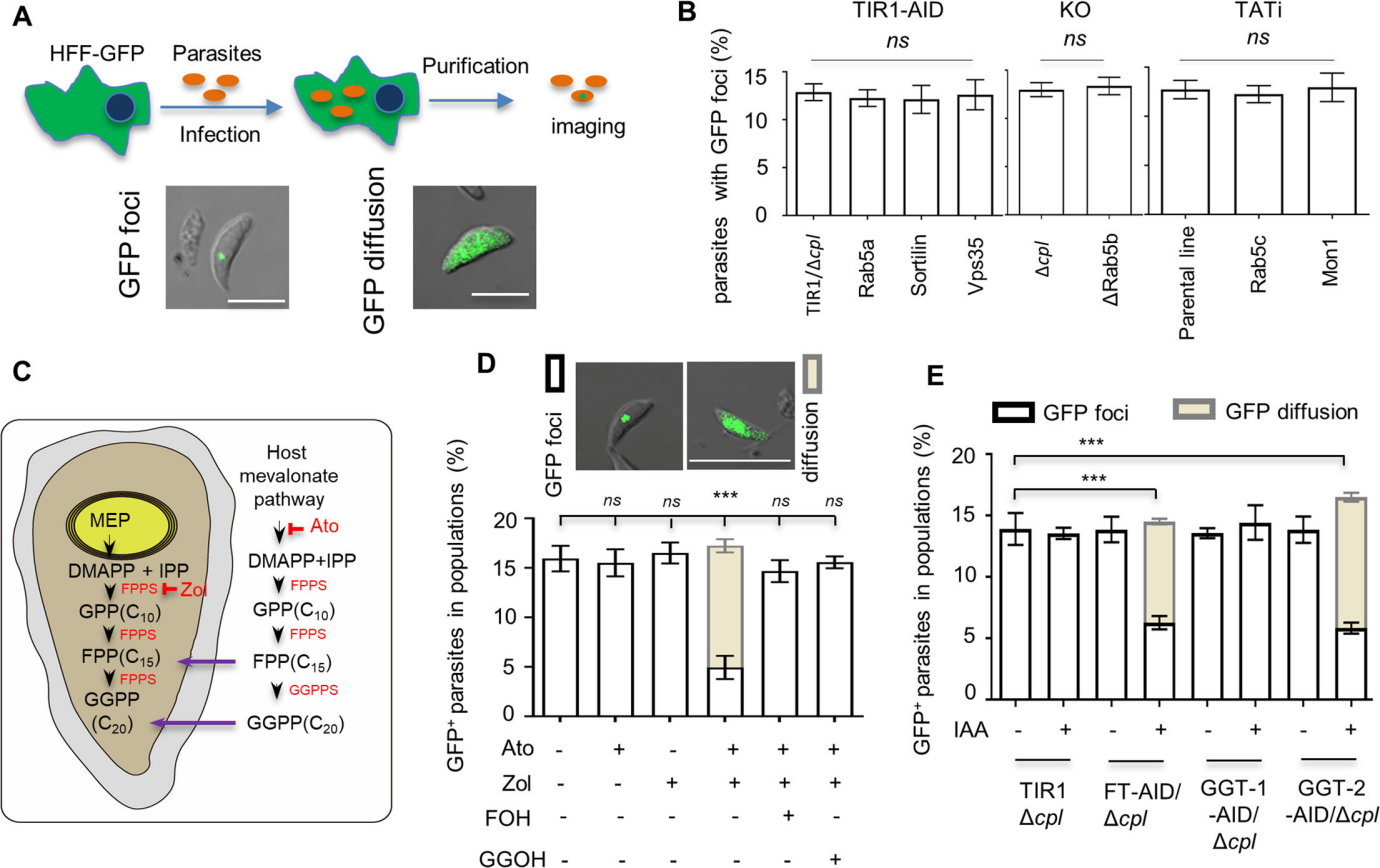
Protein prenylation is involved in endocytic trafficking in *T. gondii*. (**A**) GFP transport assay in *T. gondii*. Parasites were grown in GFP-expressing HFF cells (HFF-GFP), followed by the purification of parasites for the observation of extracellular parasites with GFP foci/GFP cytosolic accumulation or without GFP signal. LHVS (10 µM) was added to the culture with parasites that contain a cysteine protease CPL. Scale = 5 µM. (**B**) Classical endocytic proteins are not involved in the trafficking of GFP vesicles in parasites. The representative genes were genetically modified, as illustrated and diagnosed in Fig. S1A and B. The AID and knockout (KO) lines were generated in the Δ*cpl* parental lines, allowing a better examination of GFP ingestion (4). Parasites were grown in GFP-expressing HFF cells in auxin for 18 hours (the AID lines) or in ATc and LHVS for 24 hours (TATi) for imaging of parasites. Parasites on images were counted blind for scoring of parasites with GFP foci, cytosolic accumulation of GFP, or absence of GFP. (**C**) Model of isoprenoid synthesis in the parasites and host cells. The MEP (methylerythritol phosphate) and mevalonate pathways localize to the parasite apicoplast and the host cell, respectively. In *Toxoplasma gondii*, the MEP pathway can produce isopentenyl diphosphate (IPP, a basic five-carbon unit) and its allyl isomer dimethylallyl diphosphate (DMAPP) for synthesis of isoprenoids. Purple arrows show isoprenoids imported to the parasites as demonstrated previously (34). Ato, Atorvastatin; Zol, zoledronate. (**D**) Inhibition of GFP transport by atorvastatin (12.5 µM) and zoledronate (0.3 µM). Parasites (Δ*cpl*) were grown in GFP-expressing cells for 24 hours with one or both of the drugs. The prenyl of FOH (10 µM) or GGOH (5 µM) was additionally used to examine the potential complementation phenotype in the parasites treated with both drugs. Parasites (GFP+) with GFP foci or cytosolic accumulation of GFP were plotted as percentages in populations. Example images of parasites with GFP foci or cytosolic accumulation of GFP are shown. Scale = 5 µM. (**E**) Protein prenylation was involved in GFP transport. Parasites with Δ*cpl* were grown in GFP-expressing host cells in ±auxin for 18 hours. The parasites were then visualized for scoring and calculation of the percentages of GFP^+^ parasites. Three independent experiments with triplicates were performed (*n* > 100 for each replicate), and data are shown as a mean ± SD and analyzed by two-way ANOVA with Tukey’s multiple comparison. ***P < 0.0001; ns, not significant.

To clarify whether classical endocytic proteins are involved in endocytic trafficking, we utilized the plant auxin-inducible degron system (TIR1-AID) (31, 32) and the tetracycline transactivator-based inducible system (TATi) (33) to conditionally down-regulate the representative components of the pathway. To generate the parasite lines, we used a CRISPR tagging approach (31), first, to fuse an AID-Ty at the C-termini of proteins Rab5A, sortilin, and VPS35 in the TIR1-expressing line where the gene cpl was deleted (Δ*cpl*). Second, we integrated the inducible promoter 7TetO-SAG4-Ty at the upstream of the start codon of Rab5C and Mon1 in the tetracycline repressor-expressing line (TATi). The strategy is demonstrated in Fig. S1A and B, and detailed materials are provided in Table S1 through S3. The proteins were efficiently down-regulated in the parasites induced by the corresponding inducer auxin for 18 hours or ATc for 24 hours (Fig. S1C and D). Rab5B was deleted using a CRISPR approach in the Δ*ku80*Δ*cpl* parental line (Fig. S1E). Previous studies demonstrated that host cell cytosolic materials (e.g., GFP) are ingested by the parasites and then accumulate in the VAC. This phenomenon can be readily observed in Δ*cpl* parasites or parasites treated with a cathepsin protease L (CPL) inhibitor morpholinurea-leucine-homophenylalanine-vinylsulfone-phenyl (LHVS) (10 µM) (4, 5). We suspected that any defect in the trafficking steps would result in diffusion of the GFP vesicles generated by endocytosis at the micropore (3). To test the above-described proteins, the parasite lines were grown in GFP-expressing human foreskin fibroblast (HFF) cells in IAA for 18 hours or in anhydrotetracycline (ATc) for 24 hours with the addition of LHVS (10 µM), followed by the examination of extracellular parasites by microscopy (Fig. 1A). Here we refer to the assay as GFP transport. On depletion of these proteins in the assay, we did not visualize parasites manifesting GFP diffusion. Instead, we observed unchanged proportions of parasites with GFP foci in the protein-depleted parasites (Fig. 1B). We further analyzed the intensity of the GFP signal in the parasites depleted with the proteins, which showed no significant changes to the GFP fluorescence accumulated inside the parasite (Fig. S2), by comparison with the uninduced parasites. However, we are unable to completely exclude the possibility that residue proteins are able to execute the GFP transport or that these proteins do not cause diffusion of the GFP vesicle but a localization shift from the VAC, as demonstrated for VPS45 in *T. gondii* (9). Nevertheless, these results suggest that these classical proteins should have no role in the trafficking processes.

To further explore this, we postulated that protein prenylation, one of the crucial roles of isoprenoid biosynthesis in eukaryotes, might play a critical role in endocytic trafficking in *T. gondii*. Though drug inhibition studies on apicoplast isoprenoid biosynthesis have been conducted in *P. falciparum* (28, 29), there is still a lack of direct evidence supporting the role of protein prenylation in endocytic trafficking. Here we exploited the GFP transport assay system and performed a similar drug inhibition with zoledronate and atorvastatin, which inhibits the biosynthesis of FPP and GGPP in the parasite and host cells, respectively (34) (Fig. 1C). In the GFP transport assay, a parasite line with the deletion of cpl (Δ*cpl*) was utilized for a better examination of GFP signal in the parasite, as described previously (4). Using IC50 concentrations of the drugs, the assay showed cytosolic accumulation of GFP in the parasites and a significantly reduced proportion of parasites containing GFP foci (Fig. 1D). This resembles the phenotypes observed in *P. falciparum* when conducting the drug inhibition studies (28, 29). To confirm whether the defective GFP transport is due to the lack of isoprenoids used for protein prenylation (i.e., FPP/GGPP), we substituted the polyprenol analog farnesol (FOH, the alcohol analog of the prenyl group FPP) (10 µM) or geranylgeraniol (GGOH, the alcohol analog of the prenyl group GGPP) (5 µM) into the medium of the drug-treated parasites. We clearly observed that the defect of GFP transport was rescued by the addition of FOH or GGOH (Fig. 1D), supporting the role of protein prenylation on GFP transport. We further tested the effect of prenyltransferases on the GFP transport by conditional knockdown of the proteins. We identified β subunits of farnesyltransferase (FT, TGGT1_200370), geranylgeranyltransferase (GGT-1, TGGT1_278230), and Rab geranylgeranyltransferase (GGT-2, TGGT1_316270) using a homolog search, with BLAST in *T. gondii*, and generated AID lines, for each, as described in Fig. S1A. After confirming the efficacy of protein depletion (Fig. S3A), parasite growth was examined by plaque formation, which showed that depletion of GGT-1 and GGT-2 removed the parasite growth, while depletion of FT had no effect on parasite growth (Fig. S3B). The GFP transport assay showed that the proportion of parasites with GFP foci was significantly reduced, and in the meantime, the parasites with GFP diffusion in the cytosol significantly increased in the FT-AID and GGT-2-AID parasites grown in auxin for 18 hours, when compared with the parasites in the vehicle (Fig. 1E). Collectively, we conclude that protein prenylation is directly associated with the trafficking of GFP vesicles in *T. gondii*.

### Identification of the prenylome in *T. gondii*

**Fig 2:**
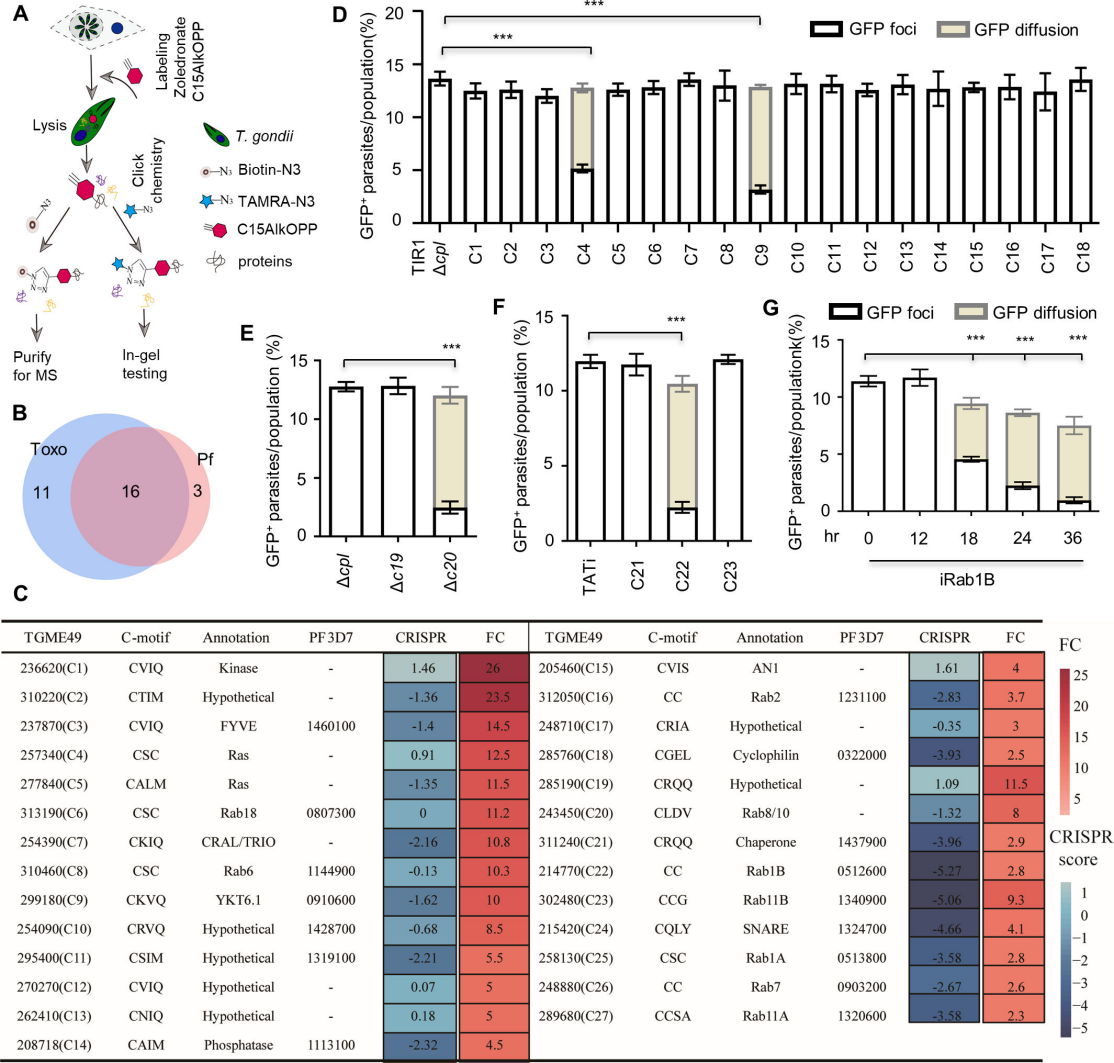
Discovery of proteins governing trafficking of endocytosed cargos in *T. gondii*. (**A**) Schematic of prenylome identification using an alkyne-labeled click chemistry approach. Parasites were grown in an excess amount of C15AlkOPP (10 µM) to substitute for the endogenous FPP and GGPP for protein prenylation. Atorvastatin was added to inhibit the host supply of FPP and GGPP. Proteins in the parasite lysate reacted with either TAMRA-N3 or biotin-N3 for in-gel testing or enrichment of prenylated proteins. (**B and C**) Comparison of the prenylated proteomics (prenylome) in *T. gondii* (Toxo) and *P. falciparum* (Pf). Shared candidates were identified in the prenylome of *T. gondii* and *P. falciparum* (**B**) (35, 38). The prenylome candidates were listed in the table with CRISPR fitness scores (39), the C-motifs, abbreviated annotations, the accession numbers, and assigned candidate numbers (C1–27) (**C**). The table includes fold changes (FC) of peptide numbers in C15AlkOPP versus FPP, and the prenylomic orthologs in *P. falciparum* (35, 38) are included for comparison. The table information was scaled to show the fold changes and CRISPR fitness scores. See details in Table S5. (**D–F**) The functional screening of prenylated candidates on GFP transport in *T. gondii* using genetic tools of the TIR1-AID system (**D**), gene knockout (**E**), and the anhydrotetracycline (ATc)-inducible expression (TATi) system (**F**). Parasites were grown in GFP-expressing cells in auxin (**D**) for 18 hours or in ATc (**F**) for 24 hours for scoring of parasites. The TIR1 lines and the knockouts harbor a deletion of cpl, while the TATi parasites containing a wild-type cpl require the addition of LHVS in the assay of GFP transport. (**G**) The GFP transport was further examined for iRab1B in ATc for 0, 12, 18, 24, and 36 hours. Parasites were counted blind for scoring parasites with GFP foci, cytosolic accumulation of GFP, and absence of GFP in parasites populations. Three independent experiments with triplicates were performed (*n* > 150 for each replicate), and data are shown as a mean ± SD and analyzed by two-way ANOVA with Tukey’s multiple comparison. ***P < 0.0001.

We then set out to identify the prenylome (prenylated proteome) in *T. gondii*. We leveraged a metabolic labeling approach in which an alkyne-labeled prenyl analog (C15AlkOPP) can be utilized to substitute for endogenous isoprenoids, including C20 GGPP, as described in previous studies (35 - 37). Proteins modified by the analog subsequently undergo click chemistry (bioconjugation) with tetracycline tetramethylcarboxyrhodamine azide (TAMRA-N3) or biotin-N3, followed by in-gel staining or enrichment of prenylated proteins (Fig. 2A). This approach has been successfully utilized to identify the prenylome in mammalian cells (36, 37) and in *P. falciparum* (35). Notably, the analog is able to transform into C20AlkOPP for another type of modification—geranylgeranyl modification of proteins (35). In the labeling experiments, atorvastatin was added to reduce the supply of endogenous FPP and GGPP from the host cells. Using the metabolic labeling and the click chemistry approach, we successfully observed additional bands on the gel staining (Fig. S3C). To analyze replicate mass spectrometry (MS) (Table S4), we used unique peptide numbers for each identified protein to calculate the enrichment of proteins (fold changes) over the samples treated with C15AlkOPP versus FPP (Table S4 and S5). As proteins containing the -CC and -CXC motifs were generally modified by GGT-2 (30), our results indicated that the analog was effectively incorporated into geranylgeranylated proteins or that it was successfully converted to C20AlkOPP for the protein modification in parasites. We analyzed the C-terminal motifs of all the candidates and identified 27 candidates in *T. gondii* (Table S5). Recently, the prenylome in *P. falciparum* has been reported (35, 38), and we then compared the orthologs of these, in both parasites, finding that 16 proteins are shared by the parasites (Fig. 2B). While the *T. gondii*-specific candidates mainly contain the -CaaX motif (Fig. 2C), the shared candidates particularly those with the -CC/-CxC motifs are likely to be functionally conserved and regulate similar biological processes in the two parasites.

### Functional screening of the prenylomic candidates

To gain insight into the cellular roles of these prenylated proteins, we functionally screened the prenylomic candidates identified in *T. gondii* and assigned the candidates the temporary names C1–27 (Fig. 2C; Table S5). Many of the candidates might be able to be deleted as suggested by the CRISPR fitness scores (39) (Fig. 2C). However, we considered that it would be still good to attempt the conditional TIR1-AID system to study the proteins since the TIR1-AID system could regulate protein levels in about 1 hour (31, 32, 40). We fused AID-Ty at the N-termini of the candidates in the TIR1/Δ*cpl* parental line to avoid interference of prenylation at the C-terminal motifs (Fig. S4A). We successfully generated 18 AID fusions (C1–C18) that were able to be depleted in the presence of auxin as demonstrated by IFA (Fig. S4B). However, the TIR1-AID system did not work for the remaining candidates (C19–C27). We then tried to construct gene knockouts for genes of C19 and C20 and made the knockout lines in Δ*ku80Δcpl* (Fig. S5A and B). We also attempted another conditional system—the TATi system, with which we generated the TATi lines for three of the other candidates (C21–C23) that were efficiently down-regulated in ATc (Fig. S5C and D). However, we were unable to generate conditional lines for the remaining candidates (C24–C27), despite several attempts being made. Thereafter, we analyzed GFP transport by growing the parasites in GFP-expressing cells, in the presence of appropriate inducers (IAA for the TIR1 derivatives or ATc for the TATi derivatives in 10-µM LHVS) for the conditional lines and by scoring extracellular parasites for GFP foci or GFP cytoplasmic accumulation in parasite populations. Our functional screening yielded positive results, where we observed dramatically reduced proportions of parasites with GFP foci in the parasite lines of C4, C9, C20, and C22, by comparison with the corresponding parental lines (Fig. 2D through F). It is noteworthy that the parasites with loss of GFP foci exhibited cytoplasmic accumulation of GFP, as shown by comparison with the parental parasites (Fig. 2D through F), suggesting that depletion of the target proteins caused defects in the trafficking steps to the VAC. The target proteins include Rab1B (C22), YKT6.1 (C9), Ras (C4), and Rab8/10-like protein (C20) (Table S6). Both Rab1B and YKT6.1 are highly conserved in the apicomplexans and are found within the group of prenylated proteins shared by *T. gondii* and *P. falciparum* (Fig. 2B and C). Intriguingly, the *P. falciparum* YKT6.1 protein has been experimentally verified *in vitro* as a prenylated protein modified at the C-terminal motif by a moiety of either farnesyl or geranygeranyl (41).

In further detailed analyses with the ATc-inducible parasites for Rab1B (here termed as iRab1B), we observed that Rab1B was substantially depleted in ATc at 18 hours and dropped to a basal level after 24–36 hours (Fig. S5D through F). Consistently, we observed that the proportion of parasites with GFP foci dropped by at least 60% and 80% in parasites induced in ATc at 18 and 24 hours, respectively (Fig. 2G), suggesting a regulatory role of Rab1B and reliability of the screening outcomes. We carefully examined images of parasites with GFP diffusion in the cytoplasm and found that GFP fluorescence perfectly diffused within the parasites, and it appeared not to be contaminants of GFP debris (Fig. S6A). To better visualize the GFP signal in parasites, we presented images of multiple parasites with GFP foci, GFP diffusion, and/or without GFP signal from different parasite lines grown either in ATc (for the TATi derivative) or in IAA (for the TIR1 derivative) (Fig. S6B). These images together with the individual parasites containing GFP signal clearly demonstrated that the parasites are able to ingest host-derived GFP and diffuse in the parasites on depletion of some key regulators, such as Rab1B and Ras (Fig. S6B). To further exclude the permeability of the parasites to large molecules in an extracellular environment, we used a live/dead cell imaging kit to detect the permeability of parasites to small molecules of dyes, as described in our recent micropore study (3). This analysis detected similar levels of parasite integrity in the parasite populations of TIR1, AID-C4, and iRab1B, which were grown in the appropriate inducers (Fig. S6D). Collectively, these results demonstrated that the diffused GFP is a protein ingested from the host cytoplasm while growing in GFP-expressing host cells.

We then examined the growth phenotypes conferred by the functional regulators, among which YKT6.1 (C9) was non-essential in *T. gondii* as shown by plaque formation (Fig. S6D). This result was also true for the Ras (C4) and Rab8/10-like protein (C20) (Fig. S6D and E). We therefore focused on Rab1B to determine its role and mechanism on vesicle trafficking in *T. gondii*, since it is essential for parasite growth as discussed below. Collectively, we have successfully identified four proteins that govern the trafficking of endocytosed cargos (e.g., GFP) in *T. gondii*.

### Localization of Rab1B and Ras in the trans-Golgi network and endosome-like compartments

**Fig 3:**
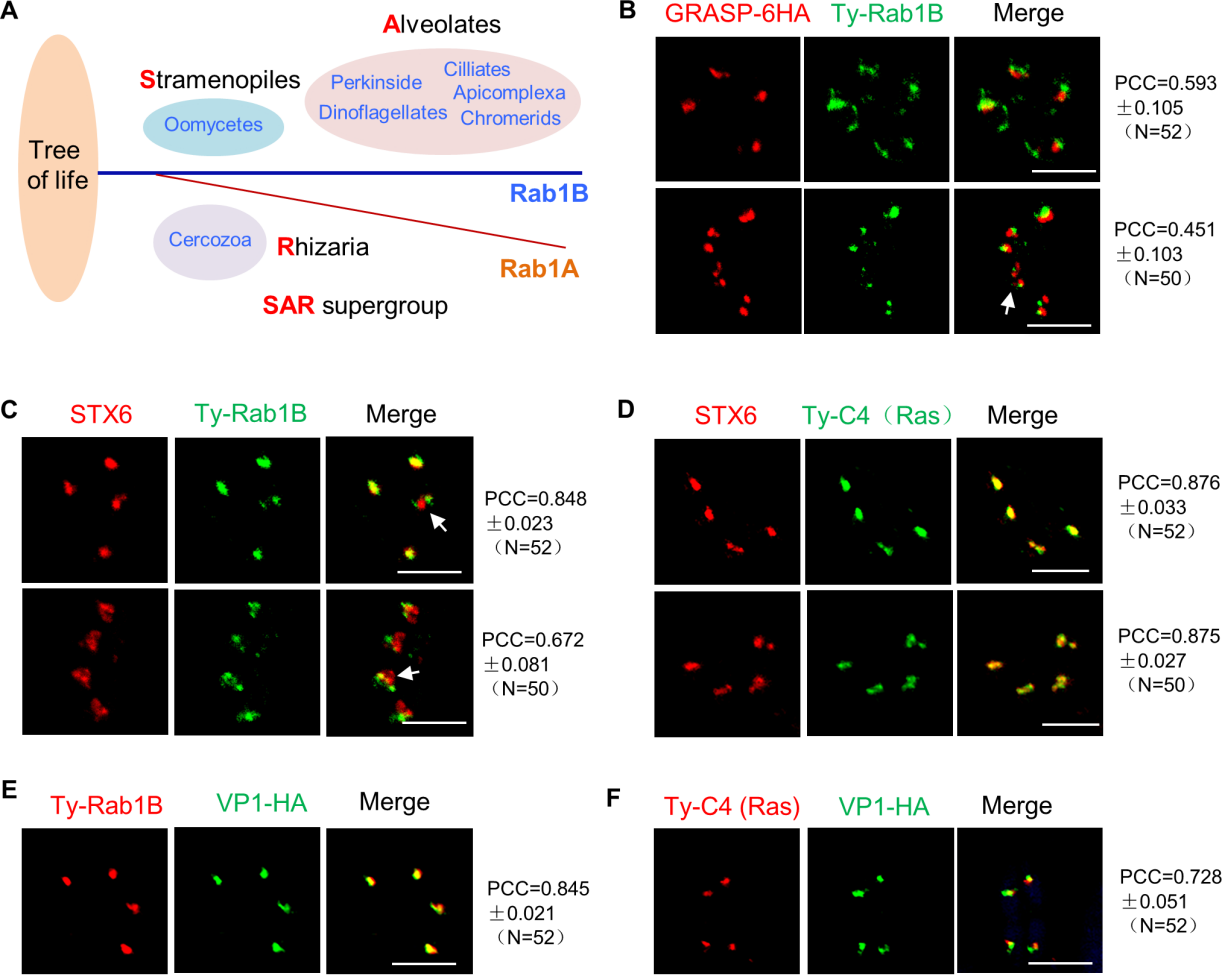
Localization of Rab1B and Ras (C4) at the trans-Golgi network and endosome-like compartments in *T. gondii*. (**A**) Schematic representation of taxa with Rab1. A unique Rab1A, the paralog of prototypical Rab1 (Rab1B in apicomplexans), was identified and shared in the recently documented SAR supergroup (42). (**B–**F) Localization of Rab1B and Ras in *T. gondii*. GRASP and VP1 were fused with HA at the protein C-terminus (for the Golgi marker GRASP and the ELC marker VP1) in the purified clones by a CRISPR approach. The parasites were analyzed by confocal imaging using mouse anti-Ty (green in B, red in E and F) and rabbit anti-HA (red in B, green in E and F) antibodies and fluorescent reagents (B, E, F). STX6 was detected by the specific antibodies that were generated in this study. Rab1B (C) and Ras (D) appeared to be one spot in the matured parasites and two foci in the dividing parasites. The fluorescent intensity was captured for the fluorescent spots for calculation of the Pearson correlation coefficient (PCC) of the protein co-localizations in the matured and dividing parasites (*N* = 50). The white arrows indicated two fluorescent foci in a single dividing parasite. Scale = 5 µM. Three independent experiments were performed, and representative images are shown (**B–**F).

Phylogenetic analysis suggested that the Rab1 subfamily has a dynamic evolutionary history (42, 43) and supported the notion that Rab1B was a prototypic protein that was vertically inherited from a common ancestor of the SAR supergroup (Stramenopiles, Alveolates, and Rhizaria) (Fig. 3A) (42). It is also notable that a paralog of Rab1B, a unique Rab1A, emerged in the SAR clade (Fig. 3A) (42). In mammals, yeast, and plants, Rab1 is implicated in endoplasmic reticulum–Golgi and intra-Golgi transport steps (44 - 48). Thus, for Rab1B in *T. gondii*, we next sought to determine its localization and functional role in vesicle trafficking, motivated by its essential role as discussed below. We endogenously fused human influenza hemagglutinin (6HA) at the C-terminus of Golgi reassembly stacking protein (GRASP), in the iRab1B-Ty line, to examine its localization relative to a Golgi marker (49). Rab1B was found to localize at the anterior of GRASP with a slight co-localization both in matured parasites and in dividing parasites (Fig. 3B). In parasites processed for co-localization analysis with STX6, it was observed that Rab1B was substantially co-localized with STX6 in matured parasites but appeared to localize at the anterior part of the STX6 compartment in dividing parasites (Fig. 3C). A previous study proposed that the STX6 compartment is the trans-Golgi network (TGN), which locates between the Golgi and endosome-like compartment (ELC) (50). We noticed that C4 (Ras) was also localized to a focus at the anterior part of parasites (Fig. S4B), and we then examined if this protein has a co-localization feature with STX6 in the Ty-C4 (Ras). Confocal imaging demonstrated that Ty-C4 was substantially co-localized with STX6. Due to the dynamic features of the TGN and the ELCs, we wondered how the proteins are localized in the parasites by comparing them with the ELC marker VP1 (51). In the parasites processed for the confocal imaging analyses, we observed a substantial co-localization of Ty-Rab1B with VP1-HA (Fig. 3E). Similarly, we observed a large part of the C4 (Ras) compartment overlapped with the ELC marker VP1 (Fig. 3F). These detailed analyses suggested that the key regulators Rab1B and Ras are substantially overlapped with both the TGN and the ELCs, supporting the intimate relation between the TGN and the ELCs (50).

We then wondered if the depletion of either Rab1B or Ras has an impact on the endosome-like vesicles (ELCs) and the TGN in the parasite. We analyzed the localization of STX6 and VP1 in the parasites by IFA on induction with the appropriate inducers. In the assay, the regular localization of STX6 was observed both in the parasite depleted with Rab1B (at 18 and 24 hours of ATc induction) and in the parasite depleted with Ras (Fig. S7A). The analysis of IFA on VP1 showed that VP1 exhibited different patterns of localization in the Rab1B-depleted parasites and C4 (Ras)-depleted parasites (Fig. S7B). Depletion of Rab1B in the parasites (in ATc for 24 hours) had no changes to the ELC marker VP1. In contrast, the depletion of Ras caused a strong localization change to VP1 (Fig. S7B and C). However, this dispersion of VP1 appeared to be different from the almost evenly distributed GFP signal in the cytoplasm of parasites while growing in GFP-expressing host cells. These results suggested that the GFP diffusion appears not to be associated with disruption of the organelles that are expected to participate in the endocytic trafficking in the parasites. Collectively, Rab1B and Ras are more likely to directly participate in the endocytic process.

### Depletion of Rab1B caused a defect in protein sorting to the rhoptry bulb

**Fig 4:**
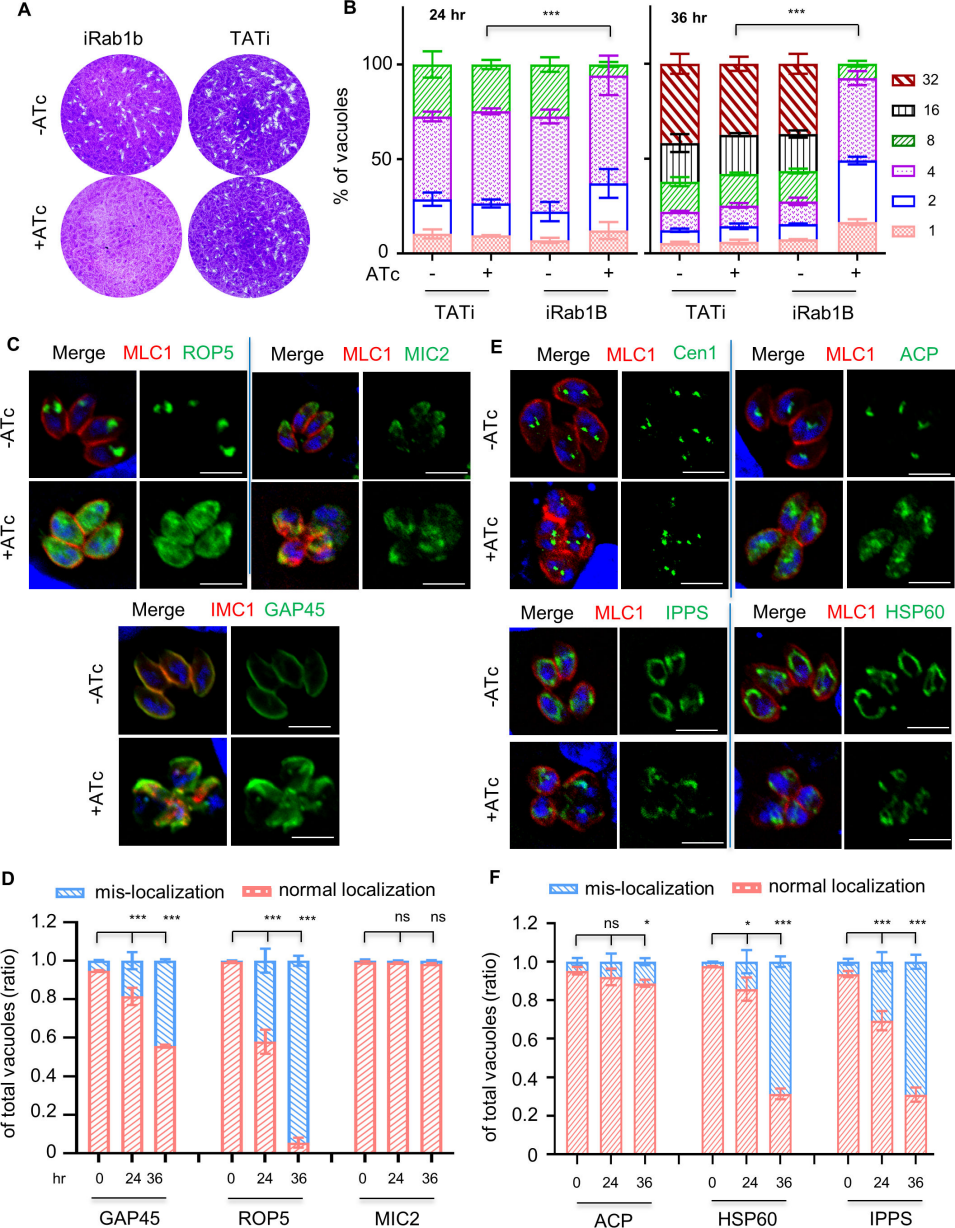
Rab1B has an effect on protein sorting to the rhoptry bulb in comparison to other cellular structures in *T. gondii*. (**A and B**) Plaque formation and parasite replication of the TATi and iRab1B-Ty lines. Parasites were grown in HFF cells in ATc (+) or ethanol (−) for 7 days (**A**), while parasite replication was examined by growth in ATc for 24 and 36 hours (hr) (**B**). IFA was performed for the scoring of vacuoles (*n* > 150) containing different numbers of parasites as described in the Materials and Methods section. In comparing iRab1B-Ty versus TATi at 24 hours, *P* = 0.0071 and *P* = 0.0003 for vacuoles containing two and eight parasites, respectively. In comparing iRab1b-Ty versus TATi at 36 hours, *P* < 0.0001 for vacuoles containing 2, 4, 16, and 32 parasites, and *P* = 0.0009 and *P* = 0.0169 for vacuoles containing 1 and 8 parasites. (**C and E**) Normal- or mis-localization of organelle marker proteins on depletion of Rab1B in *T. gondii*. Parasites were grown in ATc or ethanol for 24 hours, followed by fixation for IFA analyses using antibodies against the organelle proteins. MLC1, GAP45, and IMC1 for the inner membrane complex; MIC2 for the micronemes; ROP5 for the rhoptries; HSP60 for the mitochondrion; Cen1 for the centrioles; ACP for the apicoplast; and IPPS for the endoplasmic reticulum (ER). Scale = 5 µm. (**D and F**) Scoring of parasites with normal localization or mis-localization of protein markers for *de novo* synthesized organelles (**D**) and other organelles (**F**). Parasites were grown in ATc for 0, 24, and 36 hours, followed by IFA analyses and scoring of parasites with normal and mis-localized protein markers. At least 150 vacuoles were analyzed in each technical replicate, and the scorings were calculated and expressed as ratios in populations. Three independent experiments were performed and representative images were shown (**A, C, E**). Data are shown as a mean ± SD, and analyzed by two-way ANOVA with Tukey’s multiple comparison (**B, D, F**). *P < 0.05; ***P < 0.0001, ns, not significant.

GFP transport en route to the VAC was observed to co-localize with vesicles in transit to the micronemes in *T. gondii* (5). We speculated that Rab1B might also be involved in organelle biogenesis. Prior to the analyses, we assessed the lytic capability of cell monolayers by the parasites grown in ATc versus the vehicle. We observed that iRab1B parasites were unable to form any discernible plaques by growth in ATc for 7 days, while the parental parasites appeared normal as determined by plaque formation in ATc (Fig. 4A). Further analysis of replication showed that iRab1B parasites in ATc appeared slower than the parasites grown in the vehicle and the parental line grown in ATc at the 24-hour induction time point (Fig. 4B). In the following 12 hours of ATc induction (at 36 hours), measurements of the percentages of the parasites/vacuole showed that the iRab1B parasites had values almost identical to those of parasites at the 24-hour induction time point (Fig. 4B). This feature indicated a very strong accumulation of parasite replication defects during the induction time window of 24–36 hours. This suggests that the conditional down-regulation of Rab1B has caused a strong growth defect at later stages in the induction window.

To explore the organelle status following depletion of Rab1B, we first analyzed the rhoptries, the micronemes, and the inner membrane complex using specific antibodies against their typical markers. The apical secretory organelles are made *de novo* during daughter cell formation once per cell cycle in the M/C (mitosis/cytokinesis) phase (52, 53), and intact secretory trafficking is vital for the formation of viable daughter parasites for dissemination. These typical markers include ROP5 for the rhoptry (54), MIC2 for the micronemes (55), GAP45, IMC1, and MLC1 for the inner membrane complex (56). We observed that depletion of Rab1B by growth in ATc for 24 hours caused diffusion of ROP5 into the cytosol of parasites that appeared morphologically normal (Fig. 4C) Furthermore, it also caused a distorted IMC structure when compared with the non-induced parasites. In contrast, for MIC2, the effect was much weaker when compared to the ROP5 changes, and it was mostly present in the apical region of the Rab1B-depleted parasites, under the situation where morphology of parasites was distorted (Fig. 4C). We further measured the proportions of parasites with mis-localized proteins in the parasites grown in ATc for 0, 24, and 36 hours. From the analyses, we found that parasites grown in ATc for 24 hours had about 40% and 20% of parasites containing mis-localized ROP5 and GAP45, respectively, and the percentages further increased to 90% and 45% in parasites induced for 36 hours (Fig. 4D). In stark contrast, MIC2 retained its largely apical localization in parasites at both of its induction points (Fig. 4D). Therefore, these detailed analyses suggested to us that the secretory trafficking to the rhoptry bulb is a relatively quick effect in Rab1B-depleted parasites. This feature indicates that Rab1B is likely to be involved in the time window for the rhoptry biogenesis at the TGN compartment. Collectively, these results show that Rab1B has a role on the protein sorting to the rhoptry bulb, in comparison with the micronemes.

We next looked at other organelles: the apicoplast, the mitochondrion, the ER, and centrioles using specific antibodies against ACP (57), HSP60, cis-IPPS (decaprenyl diphosphate synthase) (58), and centrin 1, respectively. IFA analyses were conducted using the same protocol for markers for the rhoptries, micronemes, and inner membrane complex (IMC). These analyses showed that the proportions of parasites with mis-localized markers for the mitochondrion and the ER were significantly increased in parasites grown in ATc versus the vehicle for 24 hours (Fig. 4E). Contrary to that, two centrin foci (centrin 1) appeared to be normally formed and separated in the parasites (with deformed IMC structures) (Fig. 4E), suggesting that the parasite division machinery remained normal in parasites induced with ATc for 24 hours. Notably, the apicoplast (ACP) was correctly localized as judged by IFA counting assays (Fig. 4F). Further analysis of the apicoplast by ACP staining showed a similar phenotype in parasites depleted with FT, GGT-1, or GGT-2, supporting no obvious role of protein prenylation on the apicoplast (Fig. S8). Taken together with all the IFA analysis on cellular structures, Rab1B has a relatively quick effect on the protein sorting of the rhoptry bulb, though it also affects other cellular structures.

### Secretion assays confirm the defect in trafficking to the rhoptry bulb

**Fig 5:**
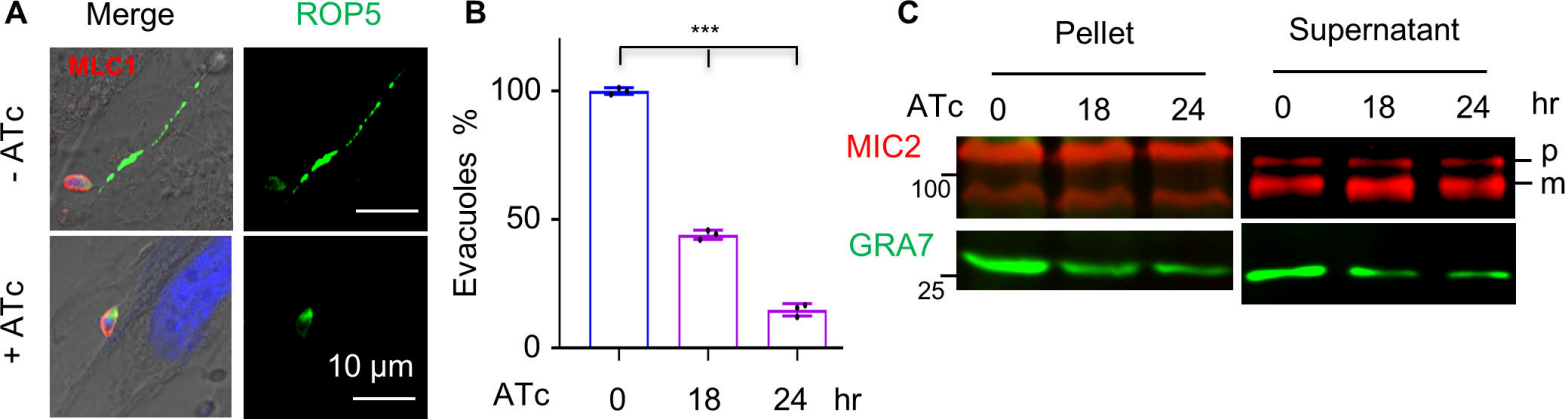
Depletion of Rab1B caused secretion defects in the rhoptry bulb but not in the microneme of *T. gondii*. (**A and B**) Defects in evacuole secretion of ROP5 in Rab1B-depleted parasites. Parasites were induced in ATc for 0, 18, and 24 hours. The parasites were then incubated in DMEM + 1 µM cytochalasin D for 10 minutes at 37°C, followed by further incubation on HFF cell monolayers at the same conditions for 20 minutes. The fixed parasites were then examined by IFA using ROP5 (green) and MLC1 (red) antibodies (**A**), and parasites with or without the ROP5 staining in HFF cells were scored (*n* > 150) and calculated for plotting (**B**). Three independent experiments with triplicates were performed, and data are shown as a mean ± SD and analyzed by one-way ANOVA with Tukey’s multiple comparison. (**C**) Western blots detected normal secretion of the micronemal protein MIC2. Parasites were induced in ATc for 0, 18, and 24 hours (hr), followed by assays of microneme secretion at 37°C in EC buffer or EC buffer with 3 µM A23187 for 10 minutes. The parasite pellets and supernatants were used for western blots using antibodies against MIC2 and GRA7. MIC2 were unprocessed (p) and processed (m) in the samples and shown on blots.

We further examined secretion from the rhoptry bulb in the iRab1B parasites. After parasite growth in ATc for 0, 18, and 24 hours, parasites were harvested and pretreated with cytochalasin D in Dulbecco’s modification of Eagle’s medium (DMEM) for 10 minutes, thus allowing the parasites to adhere to but not actually invade the HFF monolayers in a pulse invasion experiment for 5 minutes (59). The secretion of ROP5 was monitored to serve as an indicator. As expected, we observed ROP5 staining in HFF host cells for the non-induced parasites (Fig. 5A). However, we observed a defect in ROP5 secretion in the induced parasites, with the percentage of parasites with the ROP5 staining decreasing significantly after induction for 18 and 24 hours (Fig. 5B). As observed in the experiments described above, parasites were able to adhere and attach to the host cell membranes, which were attributed to the secretion of micronemes (31). We thus examined secretion of the micronemes by stimulation of parasites with A23187, which showed no obvious changes in secretion of MIC2 into the supernatant, as demonstrated by western blots (Fig. 5C). In summary, these experiments provide additional functional data supporting the effect of Rab1B on the secretory trafficking to the rhoptry bulb, but not to the micronemes in *T. gondii*.

### Protein prenylation is critical for the conserved regulation of endocytic trafficking

**Fig 6:**
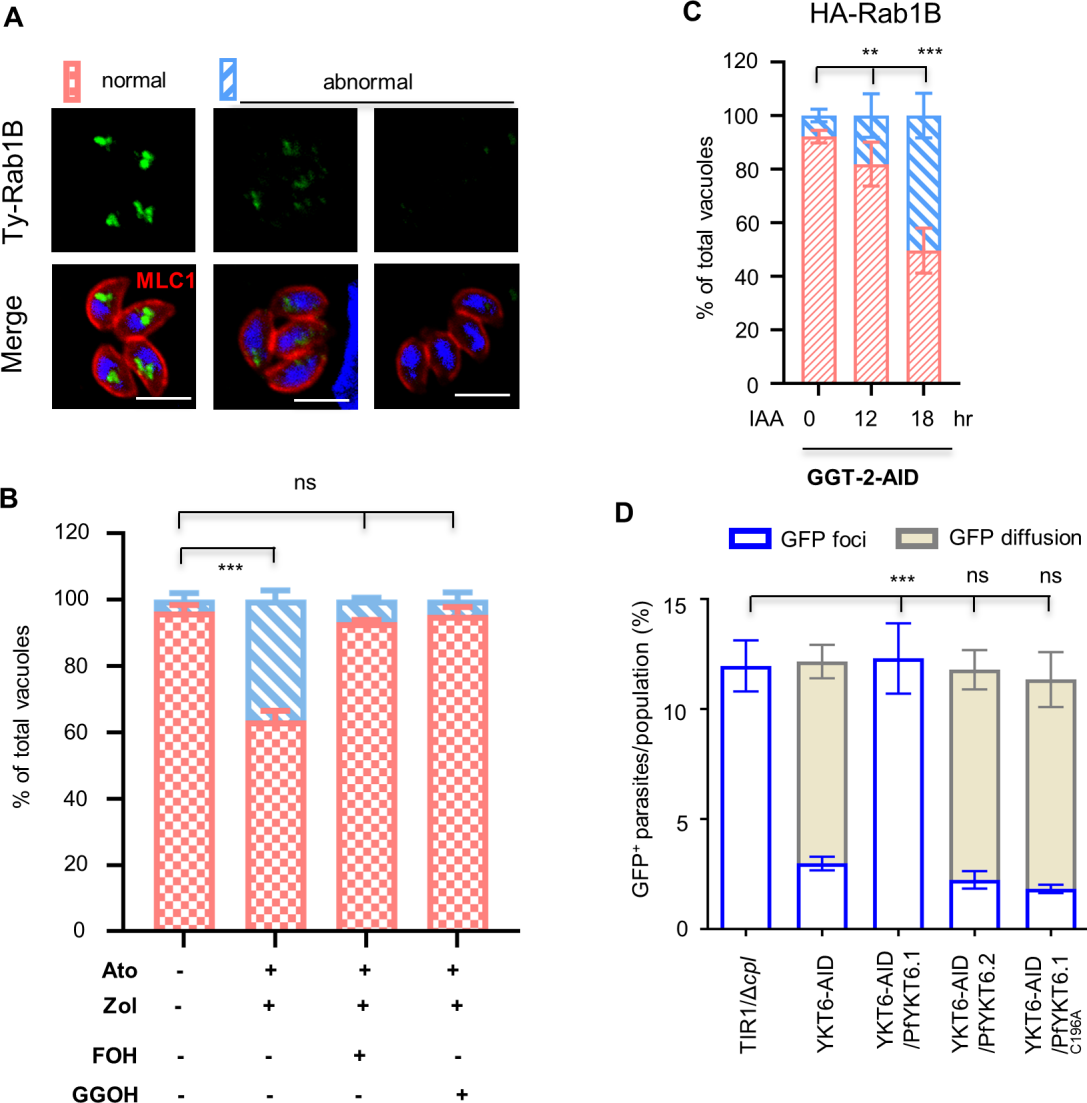
Protein prenylation is critical for the conserved regulators Rab1B and YKT6.1 in parasites. (**A–C**) Drug inhibition and depletion of GGT-2 caused a defect in Rab1B abundance in parasites. Parasites were grown at the IC50 concentration of atorvastatin (Ato) (12.5 µM) and zoledronate (Zol) (0.3 µM) for 24 hours, followed by fixation for IFA analysis of Rab1B. Parasites had an abnormal intensity of Rab1B fluorescence with either lower intensities or loss of Rab1B signal after the drug treatment (**A and B**). The parasites grown in both drugs were supplied with 10 µM FOH or 5 µM GGOH for 24 hours to examine the capability of the Rab1B phenotype in exogenously derived polyprenyl analogs (**B**). These parasites were scored and plotted as a percentage (**B**). The GGT-2-AID parasites were grown in auxin for 0, 12, and 18 hours for IFA analysis of Ty-Rab1B, and a similar defect of Rab1B was observed by the depletion of GGT-2-AID (**C**). Parasites were scored blind for the calculation of parasites with abnormal signal in the populations. At least 150 vacuoles (*n* > 150) were counted for plotting the percentages of different types of vacuoles. (**D**) Loss of the prenylation motif at the C-terminus of PfYKT6.1 caused loss of the complementation capability in the AID-TgYKT6.1 parasites. In the AID-TgYKT6.1 parasites, PfYKT6.1 and PfYKT6.2 were expressed for examination of the complementation capability of the GFP transport. The lines were grown in GFP-expressing host cells in auxin for 24 hours for scoring and calculation of extracellular parasites with GFP foci/cytosolic accumulation in the parasite population. The cysteine at the C-terminal motif was mutated to alanine (C196A) in PfYKT6.1, resulting in the loss of the complementation capability in the AID-TgYKT6.1 line. Three independent experiments with triplicates were performed, and data are shown as a mean ± SD and analyzed by one-way ANOVA with Tukey’s multiple comparison. ***P < 0.0001; ns, not significant.

We then wondered if protein prenylation would affect protein abundance and the function of the conserved proteins during endocytic trafficking. We initially attempted to complement iRab1B with Rab1B from *T. gondii* and *P. falciparum* but were unable to make the parasite lines. We then examined Rab1B in parasites by inhibition with the IC50 concentration of atorvastatin and zoledronate and by depletion of GGT-2-AID. In the drug-treated parasites, we observed a decrease or loss of Ty-Rab1B intensities (Fig. 6A). Detailed analyses demonstrated that these parasites were significantly increased in the drug-treated parasites, compared to the untreated parasites (Fig. 6B). We further substituted the poly-prenol analog FOH or GGOH into the parasite culture treated with the drugs, followed by the analysis of Rab1B as described above. We observed a rescued phenotype of Rab1B in the parasites supplied with FOH or GGOH, thus supporting the role of protein prenylation on the stability of Rab1B (Fig. 6B). Rab1B harbors a -CC motif at the C-terminus, which is predicted to be the substrate of GGT-2. We then analyzed Rab1B in the GGT-2-AID-depleted parasites by fusing Rab1B with HA at the N-terminus. Similarly, we observed significantly increased percentages of the parasites with lower Rab1B intensities, after the growth of parasites in auxin for 12 and 18 hours (Fig. 6C). It was noteworthy that ~50% of parasites retained Rab1B correctly in the GGT-2-depleted parasites. We suspect that Rab1B is able to be farnesylated and geranylgeranylated in these parasites, which was observed *in vitro* when assayed with PfYKT6.1 (41). Collectively, these data show that protein prenylation is critical for the stability and function of Rab1B in parasites.

We further examined YKT6.1 by complementation of the AID-YKT6.1 line with the ortholog of *P. falciparum*. Sequence analysis showed that both PfYKT6.1 and TgYKT6.1 harbor the same C-terminal motif (-CaaX), while PfRab1B and TgRab1B have another type of C-terminal motif (-CC) (Fig. S9A and B). To examine the complementation capability of PfYKT6.1 and PfYKT6.2, we generated the lines in the AID-TgYKT6.1 line by expression of the proteins (Fig. S9C). The expression of PfYKT6.1 appeared to complement the defective GFP transport in the TgYKT6.1-depleted parasites induced in IAA for 24 hours (Fig. 6D). In contrast, the paralog PfYKT6.2 was unable to complement the TgYKT6.1-depleted parasites, suggesting that there is functional conservation of YKT6.1 between *T. gondii* and *P. falciparum*. Intriguingly, once the cysteine was mutated to alanine (C196A) at the C-terminal motif of PfYKT6.1, it removed the complementation activity in the *T. gondii* YKT6.1 AID line (Fig. 6D). Thus, these results suggest that the conserved trafficking regulators (Rab1B and YKT6.1) require prenylation modification at these motifs for their stability and activity in the parasites.

## DISCUSSION

Apicomplexan parasites possess endocytic structures and endocytic processes (11, 60, 61), but the classical endosomal proteins are repurposed for secretory trafficking (12, 14, 62). Thus, it remains largely unknown as to how the parasites transport endocytosed cargos for digestion. Here we exploited efficient genetic tools and a host GFP acquisition assay using the apicomplexan model organism *T. gondii*, to perform a functional screen on endocytic proteins identified as prenylated candidates in this study. From this screen, we identified specific proteins (e.g., Rab1B and YKT6.1) that govern the trafficking of endocytic vesicles (e.g., those containing GFP) to the digestive vacuole. Among the proteins, Rab1B is highly conserved in apicomplexans and essential for parasite growth. Confocal microscopic analysis revealed that Rab1B and Ras (C4) are likely to localize at both the TGN and the ELCs. Detailed examination supported the involvement of Rab1B at the intersection of endocytic trafficking and secretory trafficking to the rhoptry bulb. Our further analyses in parasites provide sets of data that support the critical role of protein prenylation for the stability and function of these conserved regulators in the model organism *T. gondii*.

In *T. gondii*, endocytic trafficking has been observed to transport host cell cytosolic materials (together with GFP) to the VAC (4, 5) and maintain the stability of the parasite plasma membrane (2), following ingestion of the materials which cross both the PVM (7, 63) and the micropore plasma membrane (3). Similarly, the erythrocytic parasite, *P. falciparum,* transports hemoglobin vesicles to the food vacuoles, following the internalization of hemoglobin at the cytostome (24, 61). However, though many classical endocytic trafficking proteins are expressed in apicomplexans, these proteins appear to have been repurposed for key activities in secretory trafficking to the apical organelles—the rhoptries and micronemes—as evidenced in *T. gondii* and in *P. falciparum* (12, 14, 62, 64, 65). Here we also confirmed the lack of function of these classical proteins in the endocytic trafficking of GFP vesicles in *T. gondii*. These data therefore underscore the evolutionary divergence, from typical eukaryotic cells, of the endocytic process in these early divergent eukaryotes. To understand this process, the hemoglobin uptake, in *P. falciparum*, is very conspicuous and offers the potential to provide valuable clues. Pioneering studies proposed critical roles for protein prenylation on hemoglobin transport, as indicated by analyses of parasite physiology and biochemistry, using drug inhibition of apicoplast isoprenoid biosynthesis (28, 29). These studies hypothesized that Rab11A and Rab5 are the effectors governing the process of hemoglobin transport. Yet, these effectors were later found to participate in functions related to the biogenesis of the apical organelles and the IMC in these parasites (66, 67). In this study, utilizing approaches of drug inhibition and depletion of prenyltransferases, we directly identified an association of protein prenylation with the trafficking of endocytic vesicles in *T. gondii,* thus opening up the opportunity to tackle this difficult problem in apicomplexans.

Here we successfully identified the prenylome in *T. gondii* using an alkyne-labeled click chemistry approach which has been utilized in mammals (36, 37) and in *P. falciparum* (35, 38). Although the *T. gondii* prenylome comprises additional proteins, by comparison with the *P. falciparum* one, the conserved proteins are mostly shared with the prenylome of *P. falciparum*, suggesting that the conserved prenylated proteins are likely to regulate similar biological processes in apicomplexans, such as secretory protein sorting and parasite morphology, as shown in *T. gondii* (68). In addition, FT and GGT-2 have regulatory roles in the trafficking of GFP vesicles, indicating that the target proteins are likely to have prenylation motifs of -CaaX or -CC/CxC at the C-terminus. By leveraging the great efficiency of genetic operations and the simplicity of the GFP transport assay in *T. gondii*, our focused screen yielded four proteins, which include three small GTPases (Rab1B, Ras, and Rab8/10-like) and one SNARE (YKT6.1). Importantly, these proteins contain C-motifs of -CaaX and -CC/CxC, indicating that our screening outcomes are robust. Notably, among the proteins, Rab1B and YKT6.1 are highly conserved in apicomplexans. We noticed that both of these proteins have two paralogs in *T. gondii* and in *P. falciparum*, one of which is likely to mediate vesicle trafficking to unique intracellular compartments (41, 42). Detailed information indicated that Rab1B appears to be the prototypic ortholog in evolution. In yeast and mammals, Rab1 is involved in upstream membrane traffic, namely, ER-Golgi and intra-Golgi transport steps (44 - 48) and is required for the initiation of secretion and autophagy (69 - 71). Similarly, YKT6 is essential in ER-to Golgi vesicle trafficking and associates predominantly with the Golgi (72). It is an autophagosomal SNARE protein responsible for fusion with lysosomes (73 - 76). Only sporadic cases have shown a role for the mammalian Rab1a in early-endosome-to Golgi trafficking (77) and a regulatory role in the motility of early endosomes along the microtubules (78). Collectively, through functional screening of prenylated proteins, we have identified non-classical but conserved proteins that govern the trafficking of endocytosed cargos in *T. gondii*.

An initial study reported that the endocytosed protein is likely to transverse through the ELC, as evidenced by confocal imaging analysis (5). Our results revealed that Rab1B and Ras appear to localize to the TGN and the ELCs; however, due to the complexity of the relationship between the TGN and the ELCs, and the dynamic interaction between the organelles (50), it might not be possible to completely distinguish the organelles and localization of the proteins even with super-resolution microscopy. It is noteworthy that Ras (C4) has no obvious growth fitness on its depletion, indicating that the compartments (the TGN and the ELCs) that it possibly localizes are not affected. Indeed, depletion of Rab1B or Ras, STX6 was localized at the regular spots, suggesting that the TGN cellular structure is normal. Though the ELCs were affected by the depletion of Ras, it was normal in Rab1B-depleted parasites. Concerning the localization of the proteins, this observation is supported by another study in *P. falciparum*, where VPS45, the only classical protein that is involved in hemoglobin transport, was found to be located at the Golgi-proximal compartment in *P. falciparum* (8). Here we found that that Rab1B is apical to the Golgi marker GRASP, but substantially co-localized with the TGN marker STX6. Ultrastructural studies propose that apicomplexan parasites, similar to plants, lack early endosomes and instead have an elaborate TGN and ELCs, which are likely responsible for receiving endocytic vesicles from the plasma membrane at the micropore (50, 79, 80).

Previous observations showed that GFP vesicles could intersect with vesicle trafficking to the micronemes (5). However, our detailed examination demonstrated that depletion of Rab1B caused a defect in secretory trafficking to the rhoptry bulb, in comparison with relatively weaker and delayed defects on other cellular structures, including the micronemes. These results suggested that the primary function of Rab1B is to regulate the GFP transport and secretory trafficking to the rhoptry bulb. The phenotypic analysis of Rab1B depletion showed that both the TGN and the ELCs are not structurally affected at the early induction time points (18 and 24 hours), supporting the role of Rab1B on endocytic trafficking and secretory sorting. In addition, depletion of Ras did not result in the dispersion of the TGN and caused the dispersion of the ELC marker VP1, but in a way that is different from the GFP diffusion in the parasites. These observations thus suggested that the dispersion of the ELCs in the protein-depleted parasites is not associated with the GFP transport. Instead, our observation appeared to provide evidence that supports an alternative proposal. Small G-proteins, such as Rab or Ras proteins, are usually associated with the identity of the organelles to help membrane recognition and fusion (81). In this sense, the regulators Rab1B and Ras are likely to serve as organelle identifiers in the parasites, which then helps direct the recognition of GFP vesicles and trafficking of these vesicles either to the ELC or to the VAC. It is, therefore, expected that depletion of these proteins will cause diffusion of the GFP vesicles in the parasite cytoplasm. It would be predicted that there should be many more proteins that are involved in GFP transport. In addition, although some of the proteins and the organelles have been identified, the trafficking route of the GFP vesicles in the parasites still awaits further clarification. In this respect, there is merit in further investigation of novel proteins and the provision of a detailed mechanism that defines endocytic trafficking.

Malarial parasites rely heavily on hemoglobin endocytosed from red blood cells for their survival (24, 82 - 84). These endocytosed cargos are delivered to the digestive vacuole, generally referred to as the food vacuole in *Plasmodium* (85, 86), a process that appears to be similar to that in *T. gondii* (4 - 6). These shared features suggest a common and conserved mechanism for endocytic trafficking in *Plasmodium* and *T. gondii*. Several lines of evidence indeed support similar roles for conserved orthologs (i.e., Rab1B and YKT6.1) found in malarial parasites. First, there are similar observations in the association of protein prenylation and endocytic trafficking when using drug inhibition in *P. falciparum* (28, 29). Second, PfRab1B and PfYKT6.1 contain -CC and -CaaX motifs, respectively, which are similar to the proteins in *T. gondii*, indicating similar regulatory roles in both parasites. Third, YKT6.1 was able to fully complement the defective GFP transport in the *T. gondii* mutant. Fourth, VPS45 is involved in endocytic trafficking, in both *T. gondii* and *P. falciparum* (8, 9), indicating a functional conservation of these endocytic proteins in these parasites. Fifth, endocytosis of hemoglobin in *P. falciparum* is active in the G1/trophozoite stage (24), which at least partially shares a time window with the rhoptry biogenesis (87 - 89). This indicates that Rab1B (PF3D7_0512600) (fitness score: −3.25) is likely to regulate two biological processes in the shared time window in *P. falciparum*. In addition, the conserved protein YKT6.1 (PF3D7_0910600) has a fitness score, as low as −3.33 in *P. falciparum* (90), which is likely a result of its role in hemoglobin transport. Thus, it would be reasonable to infer that Rab1B and YKT6.1 govern the hemoglobin transport process in malarial parasites.

In summary, our work defines the molecular basis for endocytic trafficking in the model apicomplexan *T. gondii*. Here we have confirmed the participation of prenylated proteins but not classical endocytic proteins in the trafficking process. Through a focused screen of prenylome candidates identified in this study, we have revealed the role of prenylated proteins in regulating the transport of endocytosed cargos acquired from host cell cytosol and, thereby, uncovered a novel mechanism that is utilized for trafficking host cytosolic material to the digestive vacuole in these parasites. Meanwhile, the conserved protein Rab1B produces an effect on the trafficking to the rhoptry bulb, providing the evidence supporting the intersection point of endocytic trafficking with secretory trafficking in these parasites. Thus, this study contributes to current active research in developing novel anti-parasitic therapies targeted at isoprenoid biosynthesis and prenyltransferases (91 - 93). Our findings further underscore the principle that protein prenylation is essential for the trafficking of endocytosed cargos. We extend this paradigm, described in higher eukaryotes, to show that non-classical endocytic proteins govern the trafficking of endocytosed cargos in the parasites, where a unique endocytosis process takes place at the specialized cytostome/micropore. Given the highly conserved nature of prenylated proteins in apicomplexans, Rab1B and YKT6.1 are likely to control similar essential processes in related protists.

## MATERIALS AND METHODS

### Antibodies and chemicals

Primary antibodies, including rabbit anti-HA (Thermo Fisher Scientific, no. 71-5500) and mouse anti-HA (BioLegend, no. 901501) were commercially available, while primary antibodies, such as rabbit anti-ACP, rabbit anti-actin, mouse anti-IMC1, and rabbit anti-GAP45, were generated in our recent study (3). Mouse mAb 6D10 anti-MIC2 (55), rabbit anti-ROP5 (54), rabbit anti-GRA7 (94), mouse anti-Ty (BB2), mouse anti-MLC1 and rabbit anti-STX6 (generated in this study), and rabbit anti-IPPS (generated in this study) antibodies were used in the study. The secondary antibodies, including anti-mouse antibodies conjugated with Alexa Fluor (488 or 568) and anti-rabbit antibodies conjugated with Alexa Fluor (488, 350, or 568), were purchased from Thermo Fisher Scientific. Fluorescent reagents conjugated with LI-COR 680CW and 800CW were purchased from Li-COR Biotechnology. Chemicals, that is, TAMRA-PEG3-Azide (BroadPharm, BP-22479), Biotin-PEG3-azide (Sigma-Aldrich, 762024–10 mg), farnesol (FOH) (Sigma-Aldrich, F203), geranylgeraniol (GGOH) (Sigma Aldrich, G3278), 3-indoleacetic acid (IAA/auxin) (Sigma-Aldrich, no. I2886), atorvastatin (Sigma-Aldrich, no. PZ0001), zoledronic acid (Psaitong, no. Z10002), mycophenolic acid (Sigma-Aldrich, no. M3536), 6-xanthine (Sigma-Aldrich, no. X4002), pyrimethamine (Sigma-Aldrich, no. 46706), and anhydrotetracycline (ATc) (SolarBio, YZ-1035708) were commercially available. ProteoExtract Protein Precipitation Kit (Millipore, 539180-1KITCN) and BCA Protein Assay Kit (Thermo Fisher Scientific, 23227) were used for the purification of prenylated proteins.

### Parasite and host cell culture

The previously described lines RHΔ*ku80*Δ*hxgprt* (95), RHΔ*ku80*Δ*hxgprt*/TIR1 (32), RHΔ*ku80*Δ*hxgprt*/TATi-1 (96), referred to as RH^ku80^, TIR1, and TATi, were used as parental lines for the transgenic lines reported here. These lines and the derived lines (Table S1) were grown on HFF-1 cells (ATCC, SCRC-1041) in DMEM (D5 medium) supplemented with 5% of heat-inactivated fetal bovine serum, 2 mM of glutamine, and 100 units of penicillin-streptomycin at 37°C with 5% CO_2_. The parasites and HFF-1 lines were maintained free of mycoplasma using the e-Myco plus kit (Intron Biotechnology). The TIR1 and its derived AID lines were cultured in HFF with 500 µM auxin (+IAA) or 0.1% ethanol alone (−IAA) for phenotypic assays, as previously described (31, 32). The TATi and its derived inducible lines were grown in 1 µg/mL anhydrotetracycline (ATc) or 0.1% ethanol. Extracellular parasites were harvested by filtration through 3.0-micron polycarbonate membranes for phenotypic assays.

### CAS9 plasmid construction and generation of *T. gondii* lines

The pCas9-sgRNA(single guide RNA) plasmids (Table S2) were used for gene knockout and gene tagging using the strategy previously described (31, 97). Briefly, the sgRNA targeting to a specific region was selected using online prediction software as described previously (98), and the pCas9-sgRNA plasmid was constructed using a Basic Seamless Cloning and Assembly kit using the primers listed in Table S3. The existing plasmid pCas9-sgRNA (Addgene no. 54467) served as the template, and three fragments were amplified using three pairs of primers named as CAS9-F1/R1, sgRNA xx (xx denotes the gene name), and CAS9-R2, F3/R3 (Table S3). For the gene tagging, the genes encoding prenylated proteins in the functional screening were targeted to the upstream of the translational start code by sgRNA 5′, while other genes were targeted to either the upstream of the translational start code (sgRNA 5′) or the downstream of the translational stop code (sgRNA 3′). The primers containing the specific sgRNA (CAS9-sgRNA xx) were listed in Table S3 for individual genes. The generation of *T. gondii* lines (Table S1) followed the tagging strategy developed in previous studies (31, 40) and as illustrated in the corresponding figures. Briefly, the short homology regions targeting the start codon region were selected at the upstream of the 5′ sgRNA (HR1) and the start codon region (HR2). The primer containing HR1 was named as M plus the specific gene, while the primer with HR2 was named as N (N1 for AID or epitope tags and N2 for TATi lines). The short homology regions targeting the stop codon region were selected just prior to the stop codon (HR3) and the downstream of the sgRNA 3′ (HR4). The primers with HR3 and HR4 were named as L and T plus the specific genes. The pCAS9-sgRNA plasmids were able to efficiently produce CAS9 and sgRNA to create DNA double-strand breaks in parasites, which facilitates the integration of a tagging amplicon. The amplicon was generated from the generic tagging plasmids, that is, pLinker-AID-Ty-HXGPRT (Addgeneno. 86667), pNL-Ty-AID-DHFR (3), and pNL-7tetO-Ty-DHFR-LoxP (generated using primers listed) (Table S2 and S3) that contain AID or epitope tags for tagging either at the start codon region or the stop codon region using the corresponding primer pairs (M and N1/N2, or L and T). The plasmids that were used for the complementation of the TgYKT6.1-AID line were generated by cloning the gene of interest into the pNL-Ty-AID-DHFR to replace the Ty-AID region using primer pairs listed in Table S3. The point mutation in PfYKT6.1 was introduced by PCR using the plasmid containing the wild-type gene with primers listed in Table S3. The CAS9-sgRNA plasmid and the corresponding amplicon with specific homology regions were combined and transfected into recipient lines. The lines were selected by the corresponding drugs and diagnosed using the corresponding primers (Table S3) and further tested by indirect fluorescent assay (IFA). In some cases, the resistance markers were excised by transfection of pmini-Cre (99).

### Knockout generation

sgRNA targeting cpl was generated as described above using primers as listed in Table S3, while the amplicon containing hxgprt was generated from pLinker-AID-HXGPRT by the primer pair cpl-hx and T. The sgRNA and amplicon were combined and transfected into a parental line as described in the transfection method. The knockout of Rab5B, C19, and C20 was performed in a similar approach using plasmids and primers as listed in Table S2 and S3. The amplicon was generated from a plasmid (Addgene no. 70147) prepared previously in another study (97). The single clones of knockouts were checked using diagnostic PCR using primers.

### Transfection and selection of genetically modified lines

Freshly egressed tachyzoites (1 × 10^7^) were combined with the pCAS9-sgRNA plasmid (20–50 µg) and the corresponding amplicon (2–5 µg) in 200–250 µL of CytoMix buffer in a 4-mm gap BTX cuvette, following the methods in a previous study (31). The mixture was electroporated using a BTX ECM 830 electroporator (Harvard Apparatus), according to a previous study (100). Parasites were then grown in HFF monolayers and drugs were on the second day added for selection, based on the appropriate concentrations of mycophenolic acid (25 µg/mL) and 6-xanthine (50 µg/mL) or pyrimethamine (3 µM). For the transfection of pmini-Cre to remove the resistance marker, 6-thioxanthine (50 µg/mL) was applied. Days later once the selection was stable, the selection pools were sub-cloned on HFF cells in 96-well plates.

### Indirect fluorescent assay

Parasites grown in HFF monolayers on coverslips were fixed, with 4% paraformaldehyde in phosphate-buffered saline (PBS), and permeabilized in PBS containing 2.5% bovine serum albumin (BSA) and 0.25% Triton X-100. The parasites were then incubated with different combinations of primary antibodies, followed by appropriate secondary antibodies conjugated with Alexa Fluor-488 or -568. After washing three to four times with PBS containing 2.5% BSA, the coverslips were mounted with ProLong Gold Antifade Mountant with or without DAPI, and parasites were imaged using a NIKON Ni-E microscope C2+ equipped with a DS-Ri2 Microscope camera. The co-localization analysis was performed using the software NIS element AR, which is directly connected to the confocal system.

### Western blots

Freshly or mechanically egressed parasites were harvested and resuspended in PBS with 5× Laemmli sample buffer. Proteins were then separated by SDS-PAGE, followed by blotting by a BioRad wet-blotting system. Western blots were then performed with appropriate primary antibodies conjugated with LI-COR 800CW or 680CW reagents or streptavidin LI-COR 800CW for the detection of biotinylated proteins. The membranes were visualized using a Bio-Rad ChemiDOC MP imaging system.

### Antibody preparation

*T. gondii* cDNA fragments encoding full or partial polypeptides of IPPS, STX6, and MLC1, respectively, were cloned into pET28a (Thermo Fisher Scientific) using primers to create corresponding expression plasmids (Table S2 and S3). The proteins were then expressed in and purified from *Escherichia coli* BL21DE3 (Transgene Biotech) by HisPur Ni-NTA spin columns (Thermo Fisher Scientific). The recombinant proteins were prepared for anti-serum generation in mice or rabbits using Inject Freund’s complete adjuvant (Sigma-Aldrich), as recommended by the manufacturer’s protocols. The anti-sera were tested by serial dilutions on IFA and western blots, further assessed by the localization in parasites, and by molecular weights of the corresponding *T. gondii* proteins.

### Plaque formation

Both assays were performed as described in a previous study (31). Briefly, freshly lysed parasites were grown on HFF cells in 6-well plates with the addition of either inducer (500 µM auxin or 1 µM ATc) or ethanol alone (0.1%) in D5 media at 37°C for 7 days. The host cell monolayers and parasites were then fixed in 70% ethanol for 10 minutes, followed by staining with 0.5% crystal violet for 5 minutes. The plates were sequentially washed, dried at room temperature, and scanned by an HP-Scanjet G4050.

### Parasite replication

Parasites were grown on HFF in 24-well plates with coverslips in 1 µg/mL ATc for the TATi line and its derivatives or vehicle for 24 or 36 hours. The parasites and HFF monolayers were fixed with 4% paraformaldehyde for 10 minutes and permeabilized with 0.25% Triton X-100 in PBS, followed by IFA using GAP45 polyclonal antibodies and secondary antibodies conjugated with Alexa Fluor 568, and mounted by ProLong Gold Antifade Mountant without DAPI. The vacuoles (*n* > 150) containing different numbers of parasites were counted blind under a NIKON Ni-E microscope C plus. Three independent experiments with triplicates were performed, and data were expressed as percentages in the total.

### GFP transport assay

The GFP transport assay was performed as shown in a previous study (4) and in our recent study (3). The HFF line expressing GFP (HFF-GFP) was prepared using a Lentivirus expression system II in our previous study (3). The parasite lines with *Δcpl* were grown on HFF-GFP lines in a D5 medium, or the lines without *cpl* deletion were grown on HFF-GFP in the D5 medium containing 10 µM morpholinurea-leucine-homophenylalanine-vinylsulfone-phenyl (LHVS) for a time length as indicated in the study. The lines were induced by auxin or ATc, according to the background of the lines used. For drug inhibition assay in the parasites, atorvastatin (12.5 µM) and zoledronate (0.3 µM) were used to inhibit the synthesis of isoprenoids in hosts and the parasites, respectively. In the drug-inhibited parasites, the polyprenyl analog FOH or GGOH was supplied at the concentration of 10 µM and 5 µM, respectively. The parasites were cultured in HFF-GFP cells for 24 hours or a time length as indicated in detailed experiments, followed by filtration through 3.0-micron polycarbonate membranes and adherence to the poly-lysine-coated coverslips. The parasites were fixed in 4% paraformaldehyde, permeabilized in PBS with 0.25% Triton X-100, and stained in PBS with Hoechst 33258. The coverslips were mounted using ProLong Gold Antifade Mountant without DAPI, and images were captured under the same exposure parameters with NIKON C2 plus microscope. Parasites can be readily recognized with GFP foci, cytosolic accumulation of GFP signal, or absence of GFP, as demonstrated in Fig. 1A and D. Parasites were all counted blind on the same images for scoring of parasites with GFP foci, cytosolic accumulation of GFP, and absence of GFP signal. Data were calculated and plotted as percentages of parasites in populations. At least 150 parasites were counted in each technical replicate, and three independent experiments were performed.

#### Parasite integrity assay

Parasite membrane integrity was assayed using a live/dead cell imaging kit (Thermo Fisher Scientific, R37601), following the manufacturer-recommended protocol. Parasites grown in auxin for 24 hours were harvested and resuspended in DMEM, followed by mixture with the same volume of mixture of vial A and vial B. The parasites were adhered on the poly-lysine-coated coverslips, followed by an incubation for 15 minutes. The parasites were washed with PBS, and fixed, followed by visualization and imaging using a 40× objective on a Nikon Bi-E confocal microscope.

### Evacuole formation

Freshly extracellular parasites were treated with 1 µM cytochalasin D for 10 minutes and then used to challenge HFF monolayers for 20 minutes in media containing 1 µM cytochalasin D. The parasites were fixed with 4% paraformaldehyde in PBS, stained with antibodies against ROP5 and MLC1, followed by Alexa-conjugated secondary antibodies. Parasites (stained by MLC1 antibodies) with and without evacuoles (stained by ROP5 antibodies) were counted blind from at least 150 parasites per coverslip. Data were plotted as the percentage of parasites that were associated with evacuoles in total parasites scored. Three independent experiments were performed in triplicate.

### Microneme secretion assay

Microneme secretion was assayed by the incubation of extracellular parasites with calcium ionophore A23187. In brief, parasites were grown in ATc for 0, 18, and 24 hours. Parasites were purified and washed in IC buffer (142 mM KCl, 5 mM NaCl, 1 mM MgCl_2_, 5.6 mM D-glucose, 2 mM EGTA, 25 mM HEPES, pH 7.4) and then resuspended in EC buffer (2 mM KCl, 142 mM NaCl, 1 mM MgCl_2_, 1.8 mM CaCl_2_, 5.6 mM D-glucose, 25 mM HEPES, pH7.4) at a concentration of 2 × 10^8^ parasites/mL. A fraction of the parasite resuspensions was incubated with or without 3 µM A23187 at 37°C for 10 minutes, followed by centrifugation to separate the parasites from the supernatant. The supernatant and pelleted parasites were mixed with 5× SDS loading buffer and prepared for protein separation in SDS-PAGE for western blots. The blots were detected with antibodies against MIC2 and GRA7, followed by secondary antibodies conjugated with fluorescent reagents.

### Identification of the prenylome (prenylated proteins)

The experiments were performed as described previously with minor modifications (35). Briefly, the *T. gondii* RHΔ*ku80*Δ*hxgprt* line was grown on HFF cells in D5 medium in the presence of 12.5 µM atorvastatin for 24 hours. The FPP analog C15Alk-OPP (10 µM) was added for metabolic labeling of prenylated proteins, and FPP (10 µM) was used to serve as the control group. Parasites were lysed in 300-µL lysis buffer (10 mM PO_4_^3-^, 137 mM NaCl, 2.7 mM KCl, 2.4 µM phenylmethanesulfonyl fluoride (PMSF), benzonase nuclease, protease inhibitor cocktail, and 1% SDS) and sonicated for complete lysis. After centrifugation, clear supernatants (100 µg proteins) were incubated with 25 µM TAMRA-N3 in Tris(benzyltriazolylmethyl)amine (TBTA) buffer containing 1 mM Tris(2-carboxyethyl)phosphine hydrochloride (TCEP) and 1 mM CuSO_4_ at room temperature for 60 minutes. The proteins were precipitated using a ProteoExtract precipitation kit (Calbiochem) for protein separation by SDS-PAGE, and in-gel fluorescence was imaged using the rhodamine protocol embedded in a BioRad ChemiDOC MP imaging system. For enrichment of prenylated proteins, the cleared supernatants were subjected to click reactions with 100 µM biotin-N3 in a buffer containing 50 mM TCEP, 10 mM TBTA, and 50 mM CuSO_4_ for 90 minutes at room temperature. The proteins were precipitated by 1 vol chloroform, 4 volmethanol, and 3 vol PBS, and proteins were dissolved in 1% SDS in PBS buffer (1.5 mg/mL). The prenylated proteins conjugated with biotin-N3 were then purified by streptavidin beads (Pierce, 88816), followed by a protocol for purification and mass spectrometry analysis of biotinylated proteins as described previously (101). Mass spectrometry hits and peptide numbers were exported from the current view in Scaffold by settings of two or more unique peptides and 95% peptide threshold. The fold changes were analyzed by comparing the peptide numbers in the C15Alk-OPP group versus the FPP control. Candidates with -CaaX, -CC, or -CXC at the C-terminus were considered as prenylated proteins.

### Statistics

Statistics were conducted in Graphpad v8.0. One-way or two-way analysis of variance (ANOVA) with Tukey’s multiple comparison was conducted for data that fit the normal distribution, while one-way ANOVA with Dunnett’s multiple comparison was tested for data with small sizes or abnormal distributions. *P* < 0.05 was considered significant. Experiment-specific statistical information is provided in the figure legends or associated methods details including replicates (n), trials (N), and statistical tests performed.

## Data Availability

The proteomic data reported in this paper have been deposited in the OMIX, China National Center for Bioinformation/Beijing Institute of Genomics, Chinese Academy of Sciences with an accession number OMIX002068. Minimally processed data of the proteomics are available in Table S4.

## SUPPLEMENTAL MATERIAL

Supplemental Figures mbio.01309-23-s0001.docx Figures S1-S9.

Supplemental Tables mbio.01309-23-s0002.xlsx Tables S1-S6.

ASM does not own the copyrights to Supplemental Material that may be linked to, or accessed through, an article. The authors have granted ASM a non-exclusive, world-wide license to publish the Supplemental Material files. Please contact the corresponding author directly for reuse.
