# Supplementary material for: Functional screening reveals Toxoplasma prenylated proteins required for endocytic trafficking and rhoptry protein sorting
Source: mBio. 2023 Aug 7;14(4):e01309-23. doi: 10.1128/mbio.01309-23 (PMC10470541; doi:10.1128/mbio.01309-23)
Supplement: Supplemental Figures — Figures S1-S9. [file mbio.01309-23-s0001.docx]

**Functional Screening Reveals *Toxoplasma* Prenylated Proteins Required for Endocytic Trafficking and Rhoptry Protein Sorting**

**Qiang-Qiang Wang^a,b,1^, Ming Sun^a,b,1^, Tao Tang^a,b,1^, De-Hua Lai^c,1^, Jing Liu^a,b^, Sanjay Maity^d^, Kai He^a,b^, Xi-Ting Wu^a,b^, Jiong Yang^c^, Yue-Bao Li^a,b^, Xiao-Yan Tang^a,b^, Hui-Yong Ding^a,b^, Geoff Hide^f^, Mark Distefano^d^, Zhao-Rong Lun^c^, Xing-Quan Zhu^e^ and Shaojun Long^a,b,#^**

^a^National Key Laboratory of Veterinary Public Health Security and College of Veterinary Medicine, China Agricultural University, Beijing 100193, China

^b^MOE Key Laboratory of Gene Function and Regulation, National Animal Protozoa Laboratory and School of Veterinary Medicine, China Agricultural University, Beijing 100193, China

^c^State Key Laboratory of Biocontrol, School of Life Sciences, Sun Yat-sen University, Guangzhou 510275, China

^d^Department of Medicinal Chemistry, and Department of Chemistry, University of Minnesota, Minneapolis, MN 55455, USA

^e^College of Veterinary Medicine, Shanxi Agricultural University, Taigu 030801, Shanxi Province, China

^f^Biomedical Research and Innovation Centre and Environmental Research and Innovation Centre, School of Science, Engineering and Environment, University of Salford, Salford, M5 4WT, UK

**This file includes Fig S1-9 and the Legends**

**
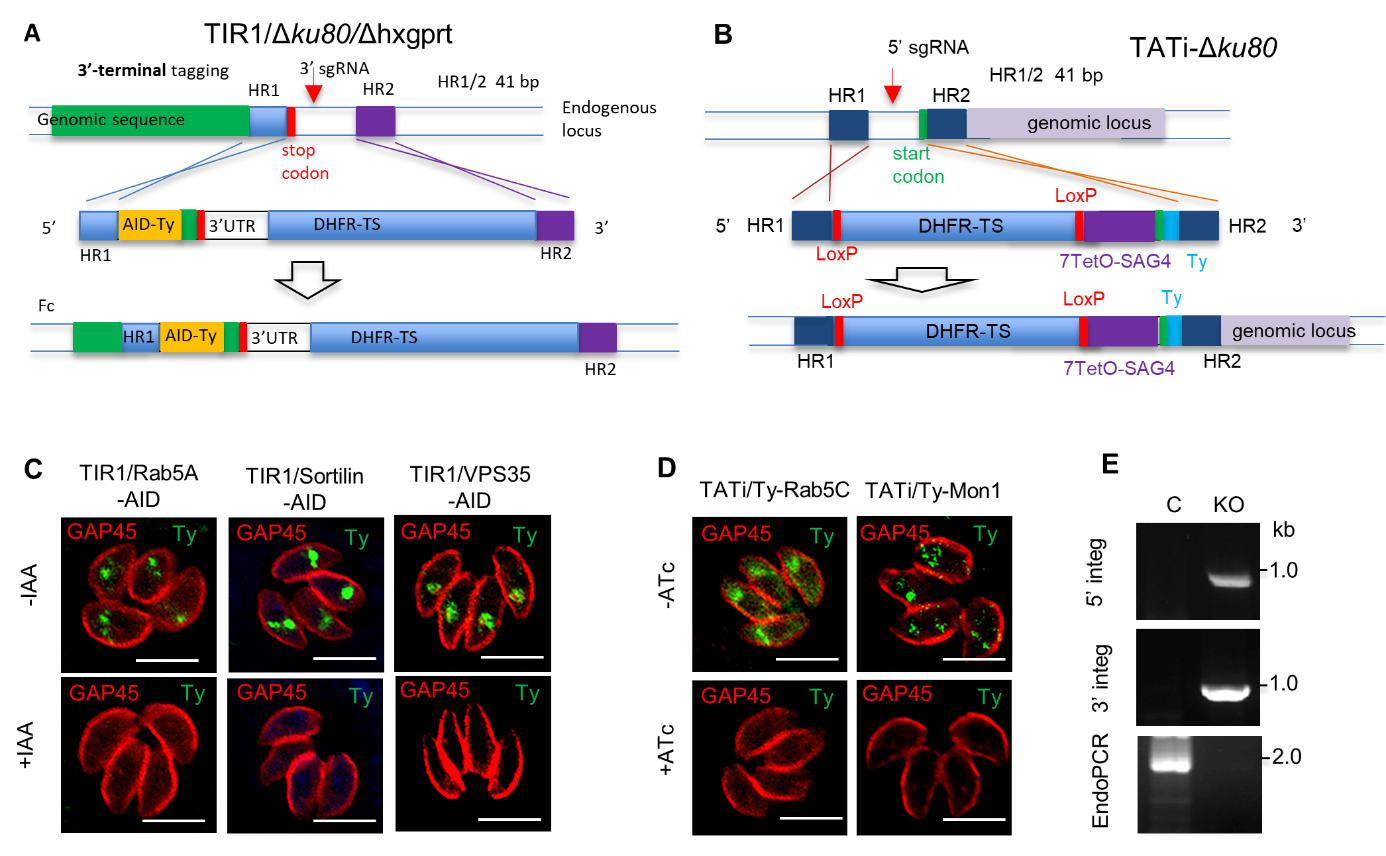
**

**Fig S1. Generation of strains for classical endocytic proteins in *T. gondii*.**

(A-B) Schematic illustration of strain generation in the parental lines of TIR1 and TATi. The AID degron was tagged at the C-terminus of a gene of interest in the TIR1 (A), while 7TetO-SAG4-Ty was added in front of the start codon of a gene of interest in the TATi (B). The genetic operation was carried out using a CRISPR-Cas9 approach, as described in our previous studies (1-3). Homologous regions (HR1 and HR2) (41 bp) were incorporated into the amplicon by PCR, which was targeted into the gene of interest by a specific sgRNA (3’ or 5’) expressed from a CRISPR/Cas9 plasmid.

(C-E) Verification of conditional knockdowns and gene knockouts. The TIR1-AID and TATi systems in combination of a CRISPR tagging technology were utilized for generation of conditional knockdowns, creating TIR1/Rab5A-AID, TIR1/SortLR-AID-Ty, TIR1/VPS35-AID-Ty, TATi/iRab5C and TATi/iMon1. The TIR1 derivative lines were grown in ± auxin for 18 hours (C), while the TATi derivative lines were induced in ± ATc for 24 hours (D), followed by IFA using primary antibodies against GAP45 (red) and Ty (green). Another gene Rab5B was able to be deleted by a CRISPR approach, as described previously (4), and diagnosed by PCR using primer pairs for amplification of 5’ integration (5’ integ) and 3’ integration (3’ integ) of the foreign DNA fragment, and another pair for the endogenous DNA fragment (endoPCR) (E). C, control, the parental line; KO, knockout; Scale bar = 5 μm.


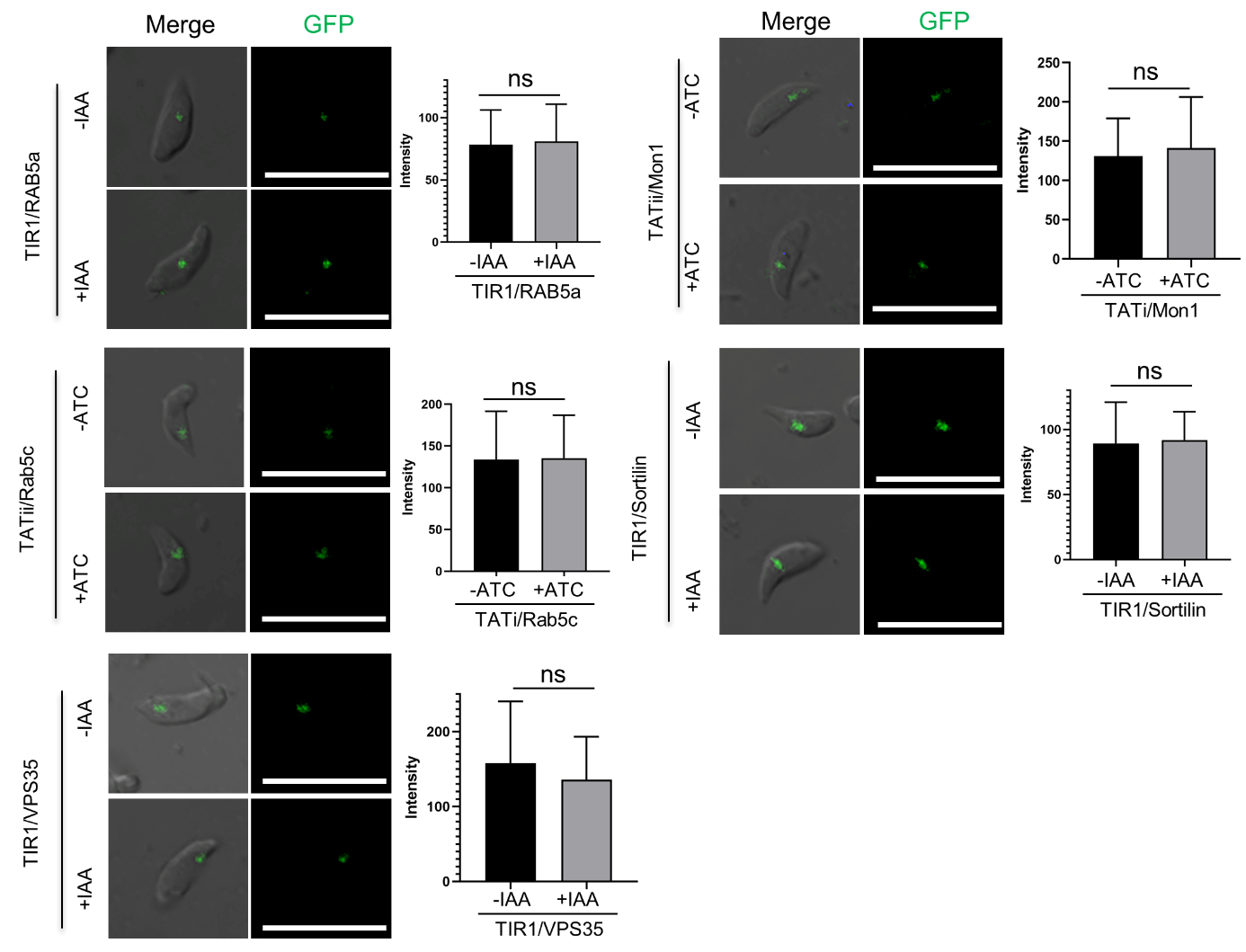


**Fig S2. Classical endocytic proteins are not involved in the GFP transport in *T. gondii*.** The parasites were grown in IAA for 18 hours (for TIR1 derivative lines) or in ATc for 24 hours (for TATi derivative lines) in GFP expressing host cells HFF. Extracellular parasites were imaged with the same parameters for scoring of the GFP intensity (N=30). Three independent experiments were performed with triplicates, and data were analyzed by one-way ANOVA with Tukey’s multiple comparisons.


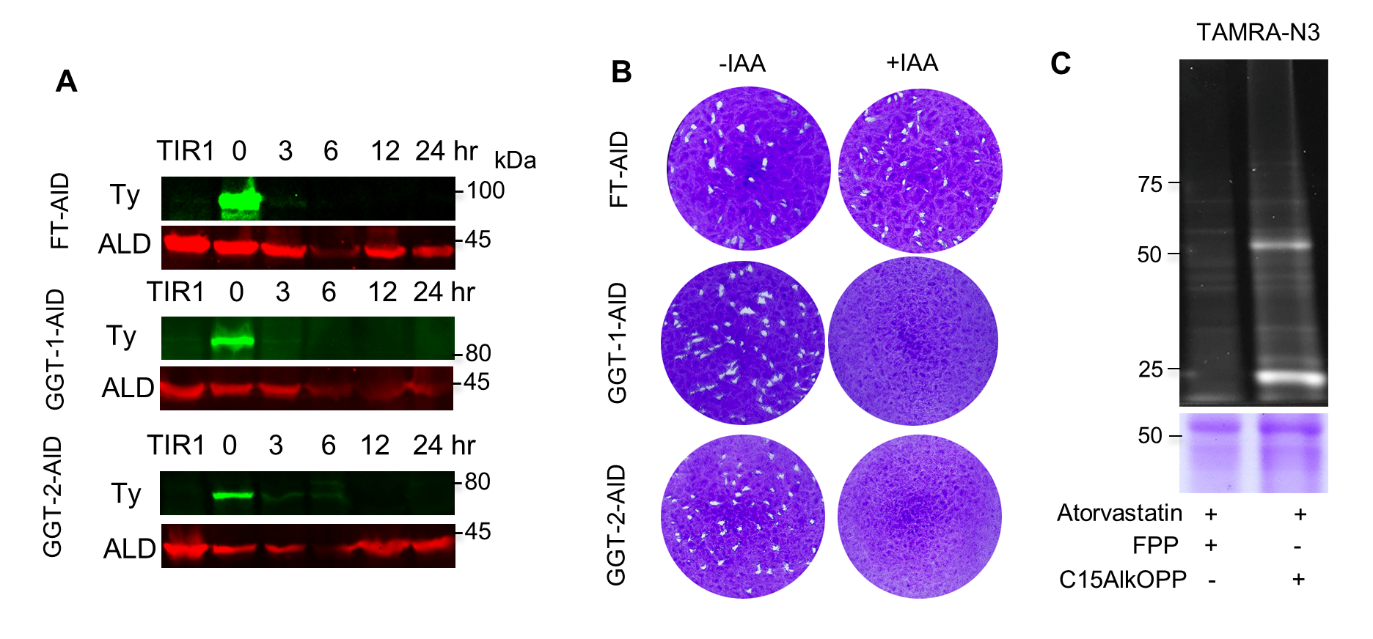


**Fig S3. Generation of AID lines for the prenyl-transferases.** The genes were fused with AID in the parental line TIR1, followed by examination by Western blots (A) and plaque formation (B). Testing of the alkyne-labeled click chemistry using an in-gel stain fluorescence technique. Parasites were grown in 10 μM analogue probe C15AlkOPP (C15) or farnesyl pyrophosphate (FPP) in the presence of the host mevalonate pathway inhibitor atorvastatin (Ato) (10 μM) for 24 hours. The parasite lysis was incubated with TAMRA-N3, allowing the click chemistry to label the analogue-coupled proteins, followed by testing with in-gel stain fluorescence (C). Total protein loading by Coomassie blue stain is shown in purple.

Three independent experiments were performed with similar outcomes.


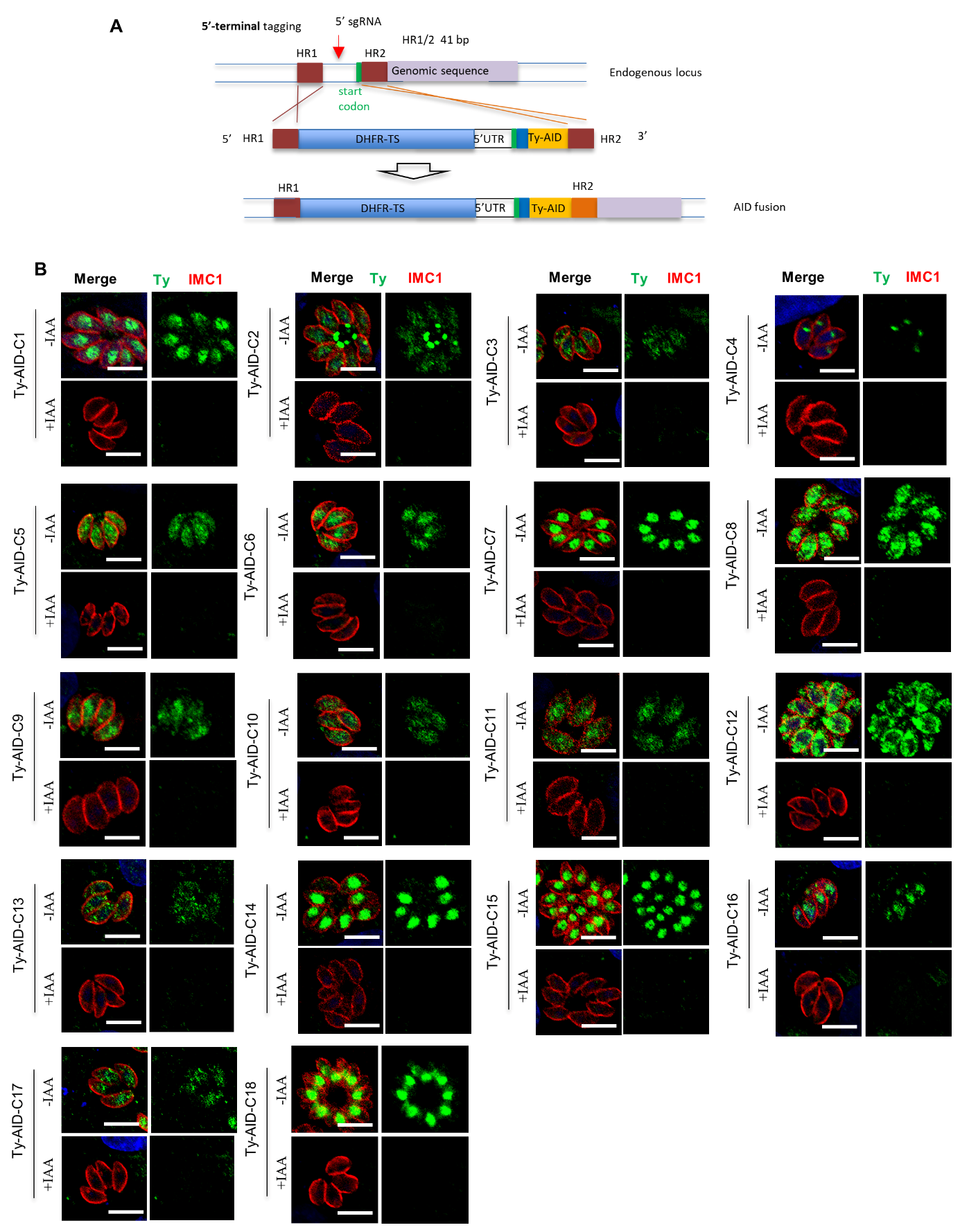


**Fig S4. Generation and verification of AID lines for prenylated proteins C1-18 in *T. gondii*.**

(A) Schematic of N-terminal tagging of Ty-AID for the candidates. The sgRNA was selected right upstream of the start codon and incorporated into pCas9 plasmid using a seamless DNA assembly kit, while the homologs regions (HR1 and HR2) were selected upstream of the Cas9 cleavage site and downstream of the start codon. The HR1 and HR2 were incorporated into the primers for amplicon generation from a generic plasmid that contains Ty-AID at the 3’ terminus. The pCas9-sgRNA and amplicon were transfected into the TIR1 parental line, resulting in the Ty-AID fragment fusion at the 5’-terminus of the gene.

(B) Efficient depletion of the AID fusions in the AID lines. Parasites were grown in auxin or ethanol for 18 hours, followed by fixation for IFA analyses using antibodies against IMC1 (red) and Ty (green). Three independent experiments were performed with similar outcomes. Scale = 5 μm.


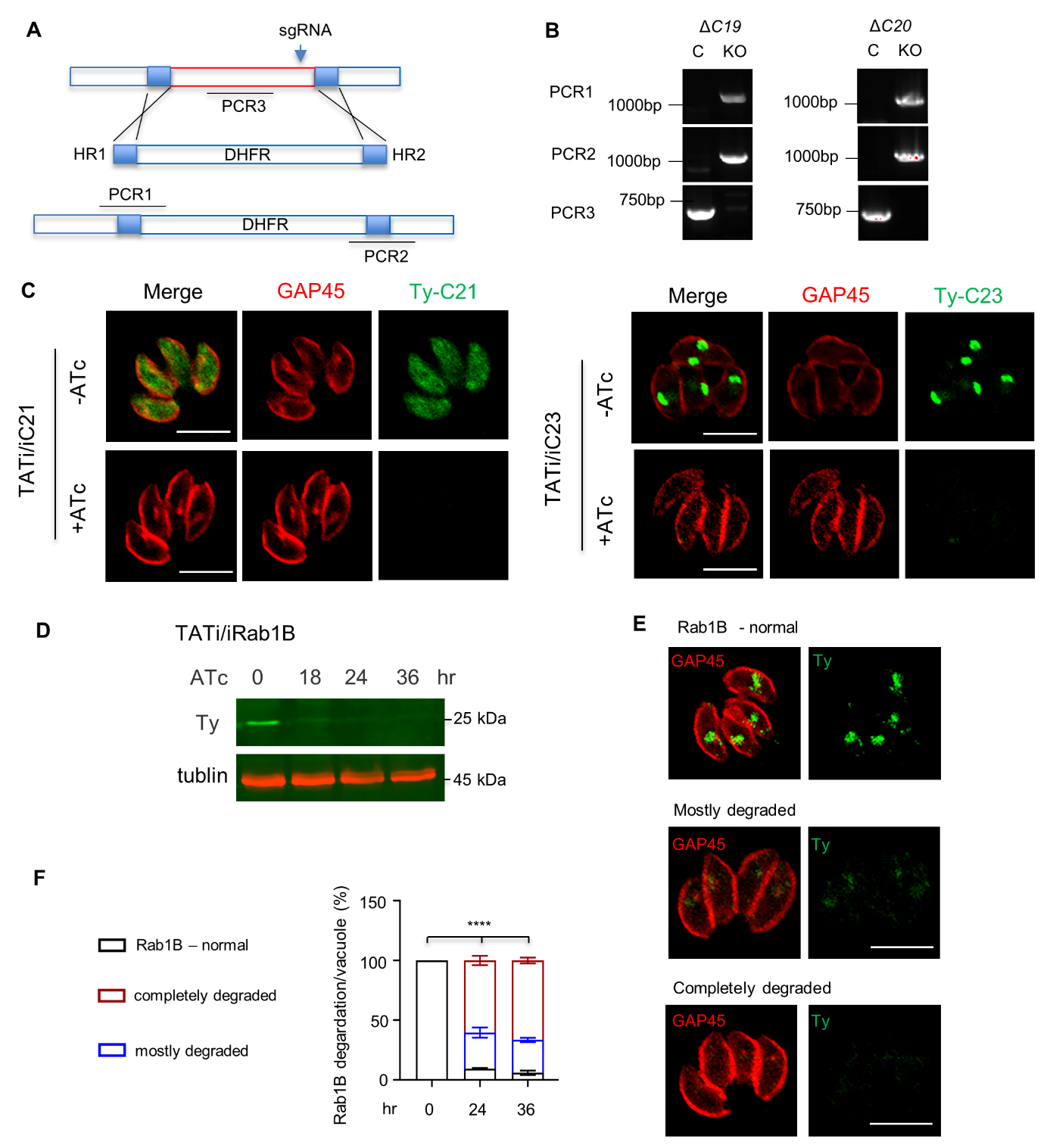


**Fig S5. Generation and verification of knockout lines and TATi derivative lines for candidates C19-23 in *T. gondii*.**

(A-B) Candidates C19 and C20 were deleted using a CRISPR editing approach. PCRs were designed for testing DNA integration at both termini (PCR1 and PCR2) and loss of endogenous genes (PCR3), as illustrated in the schematic. C, control with the parental line; KO, knockout for the corresponding genes.

(C) Verification of TATi/iC21 and TATi/iC23 by IFA. The lines were generated using the strategy illustrated in Fig S1B. Parasites were grown in ATc for 24 hours, followed by fixation for IFA analyses using antibodies against GAP45 (red) and Ty (green).

(D-F) Verification of TATi/iTgRab1B (C22). Western blots detected downregulation of Rab1B in ATc for 18, 24 and 36 hours, and tubulin served as the loading control (D). Rab1B degradation was also examined by IFA for parasites induced in ATc for 24 hours, and the results were classified into three groups: Rab1B mostly degraded, completely degraded or Rab1B normally (E). The Rab1B groups were examined for parasites induced in ATc for 0, 24 and 36 hours (F).

Three independent experiments were performed with similar outcomes (B-E). Data were shown with mean ± SD and analyzed by one-way ANOVA with Tukey’s multiple comparisons, ***, *p*<0.0001.


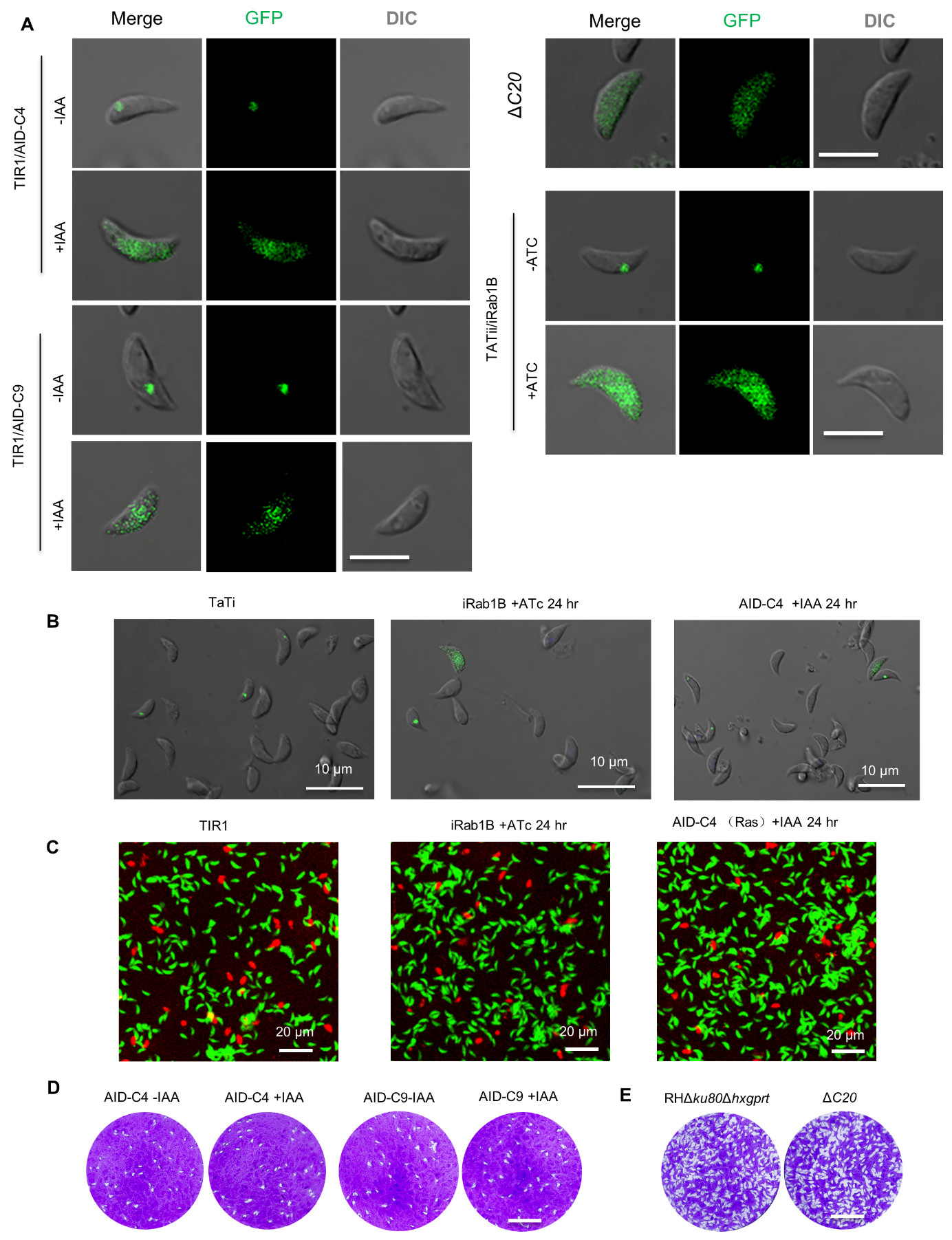


**Fig S6. GFP transport was regulated by key proteins of C4, C9, C20 and C22 (Rab1B) in *T. gondii***. (A) Parasites were grown in GFP expressing HFF cells in IAA for 18 hours (for TIR1 derivatives) or in ATc for 24 hours (for TATi derivative), followed by imaging and scoring of parasites with GFP foci or GFP diffusion. (B) GFP visualization in extracellular parasites that were grown in GFP expressing host cells. Parasites were grown in GFP expressing HFF cells in ATc (for TATi and iRab1B) or in IAA (for AID-C4) for 24 hours, followed by imaging of parasites with GFP signal. Images with multiple parasites were shown for presentation of parasites with GFP foci, GFP diffusion or without GFP signal. Scale = 10 μm. (C) Parasite permeability to the live/dead cell imaging dyes. iRab1B parasites were grown in ATc for 24 hours, while AID-C4 parasites were grown in IAA for 24 hours. The parasites were analyzed by staining with dyes from the live/dead cell imaging kit, followed by visualization under 40 objective lens in a NIKON microscope Ni-E2. (D-E) Plaque formation was examined for parasite lines of candidates C4, C9 and C20. The parasites were grown in HFF cells for 7 days, followed by fixation with 70% ethanol for staining with crystal vial. The AID parasites (150 parasites) were incubated in ± IAA, while the C20 parasite and its parental line (500 parasites) was grown directly without addition of inducer. Scale = 1 cm.


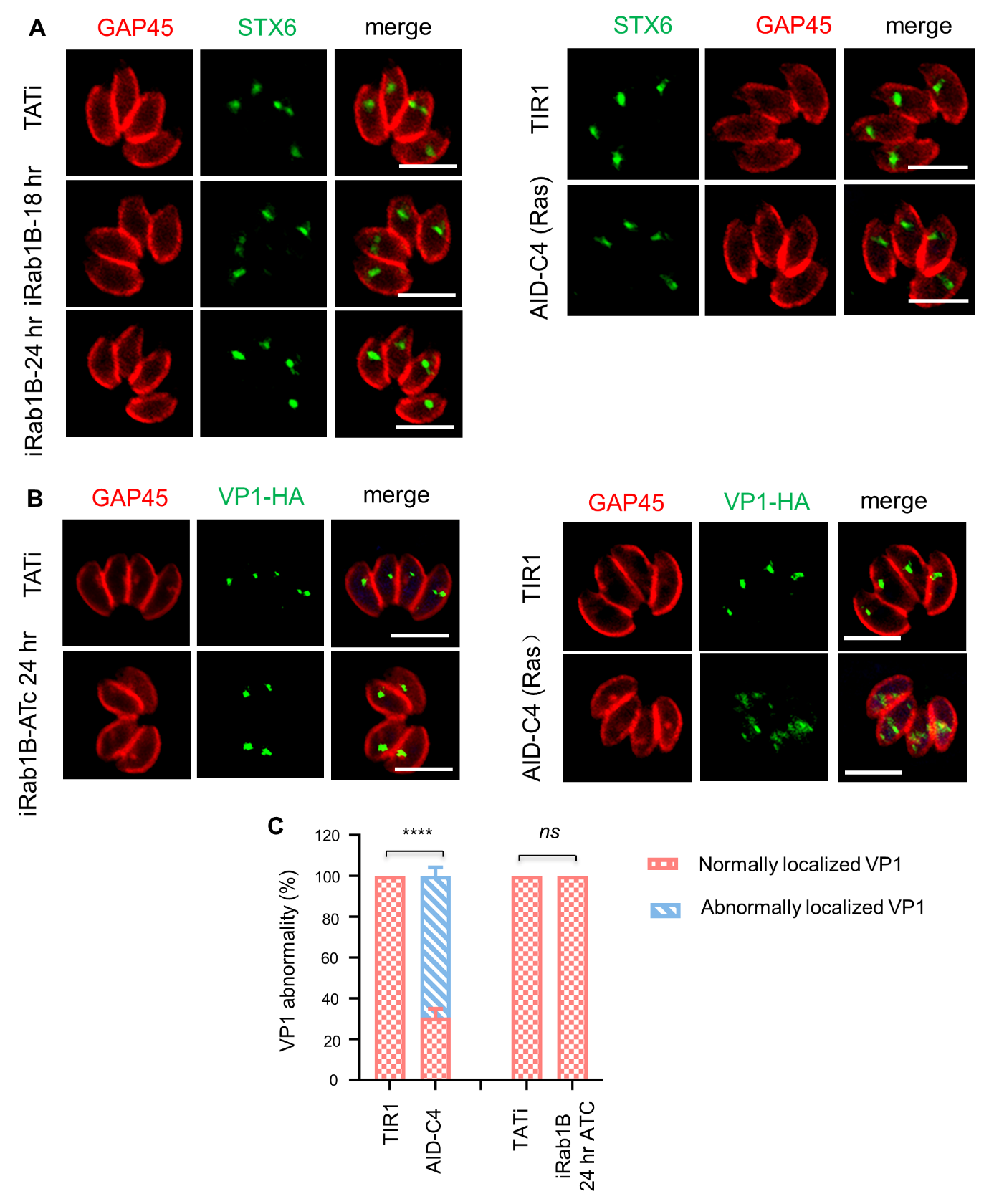


**Fig S7**. Impact on the TGN and the ELCs upon depletion of Rab1B and Ras (C4) in the parasite. iRab1B and its parental line (TATi) parasites were grown in ATc for 0, 18 or 24 hours, while TIR1 and AID-C4 (Ras) parasites were grown in IAA for 24 hours. Parasites were analyzed by IFA using antibodies to detect STX6 (A) or VP1-HA (B) for observation of their corresponding organelles. GAP45 served as the IFA control. VP1-HA was observed to be dispersed in the AID-C4 (Ras). (C) Parasites with normally localized VP1 and abnormally localized VP1 were scored in all the parasites described in (B) (n>100 parasites). Three independent experiments were performed with triplicates. Data were analyzed by one way ANOVA with Tukey’s multiple comparison. Scale = 5 μm.


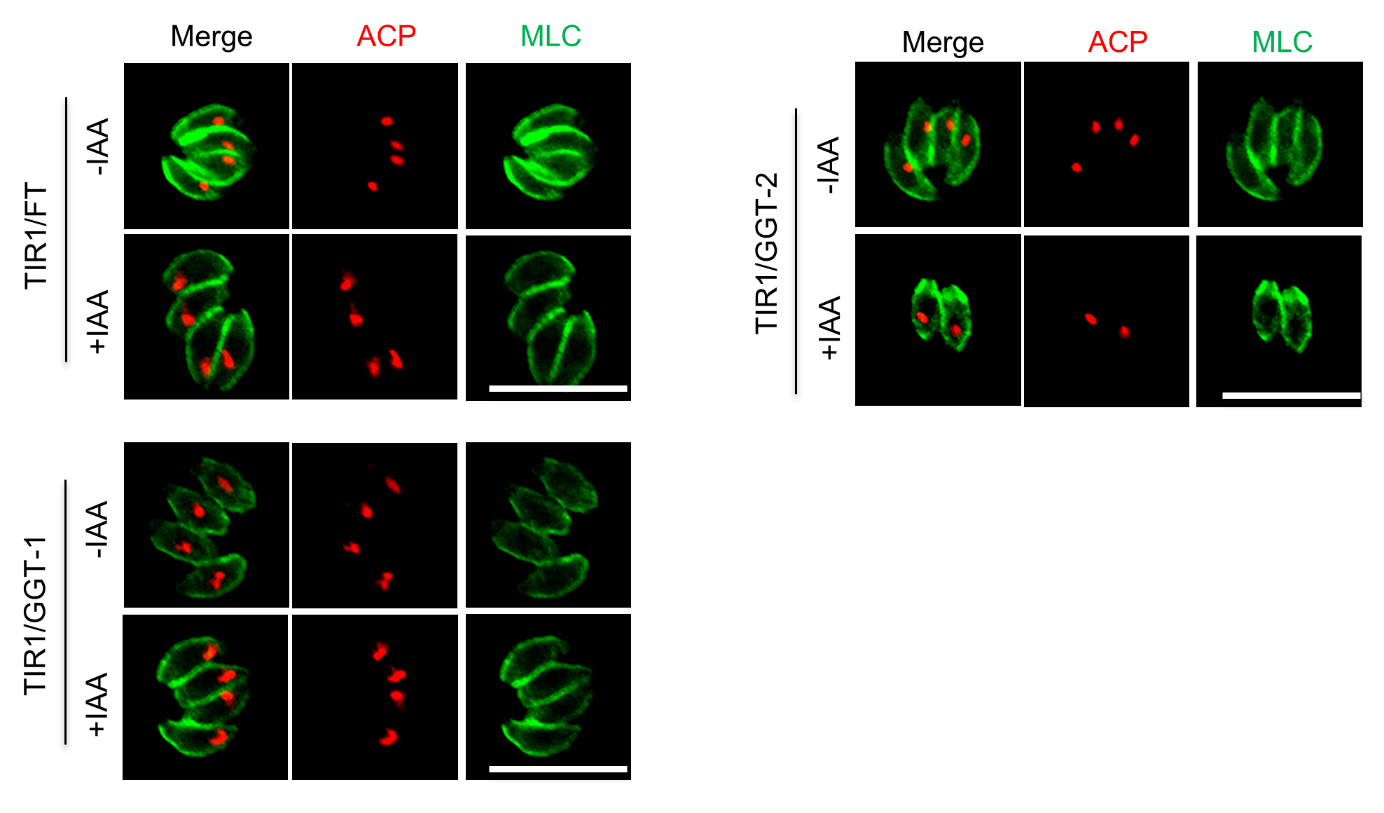


**Fig S8. The apicoplast was not affected by depletion of the prenyl-transferases in *T. gondii*.** The parasites were grown in IAA for 18 hours, followed by examination of the apicoplast status using an ACP stain.


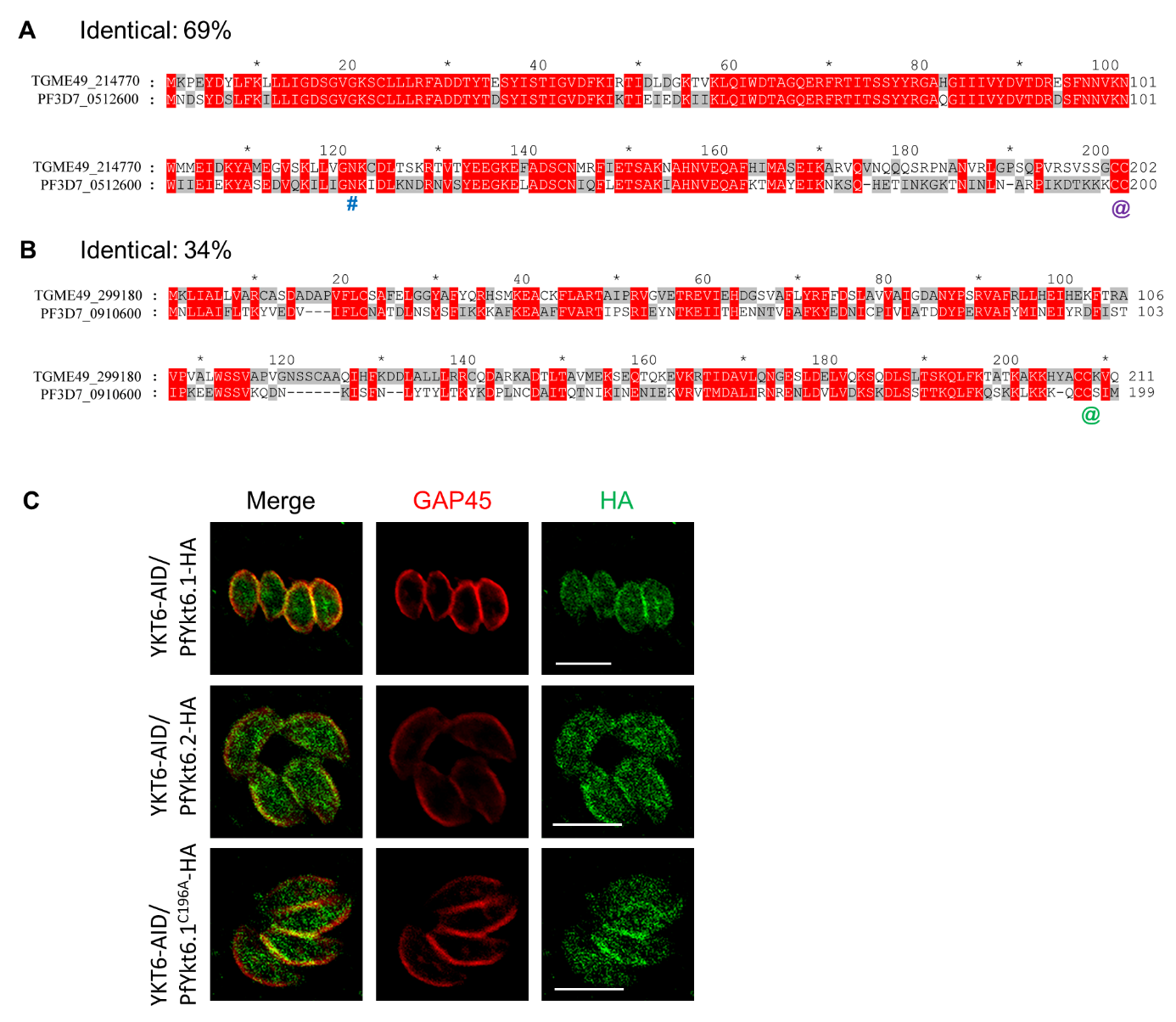


**Fig S9. Sequence alignment of Rab1B and YKT6.1 and complementation of YKT6.1 in parasites**

(A-B) Protein sequences were retrieved from TOXODB and PlasmoDB for Rab1B (A) and YKT6.1 (B), and aligned using Clustal W. Rab1B from *T. gondii* and *P. falciparum* harbors the key residue N at the 121 position, as denoted by #. Rab1B is predicted to be modified by geranylgeranyl prenyl at the C-motif of –CC, while YKT6.1 has a C-motif of –CxxA. The motifs in Rab1B and YKT6.1 are indicated by a purple and green @, respectively. The percentages of identical amino acids are shown for the proteins by comparing the *T. gondii* and the *P. falciparum*.

(C) Complementation of TgYKT6.1-AID by TgYKT6.1 and PfYKT6.1 in parasites. The TgYKT6.1, PfYKT6.1 and PfYKT6.2 were expressed in the line of TgYKT6.1-AID line, respectively for examination of complementation capability. The cysteine residue at the CxxA motif was mutated to alanine at the PfYKT6.1 for testing the complementation capability in the AID line. Parasites were grown in HFF cells for IFA analysis. Scale = 5 μM.

1. Long S, Brown KM, Drewry LL, Anthony B, Phan IQH, Sibley LD. Calmodulin-like proteins localized to the conoid regulate motility and cell invasion by Toxoplasma gondii. PLoS Pathog. 2017;13(5):e1006379.

2. Brown KM, Long S, Sibley LD. Conditional Knockdown of Proteins Using Auxin-inducible Degron (AID) Fusions in Toxoplasma gondii. Bio Protoc. 2018;8(4).

3. Brown KM, Long S, Sibley LD. Plasma Membrane Association by N-Acylation Governs PKG Function in Toxoplasma gondii. mBio. 2017;8(3).

4. Long S, Wang Q, Sibley LD. Analysis of Noncanonical Calcium-Dependent Protein Kinases in Toxoplasma gondii by Targeted Gene Deletion Using CRISPR/Cas9. Infect Immun. 2016;84(5):1262-73.
